# Supplementary material for: Visualizing a single wavefront dislocation induced by orbital angular momentum in graphene
Source: Nat Commun. 2024 Apr 26;15:3546. doi: 10.1038/s41467-024-47756-w (PMC11053005; doi:10.1038/s41467-024-47756-w)
Supplement: Supplementary file 1 — Supplementary Information [file 41467_2024_47756_MOESM1_ESM.pdf]

Supplementary Information

Visualizing a single wavefront dislocation induced by orbital angular momentum in graphene

Yi-Wen Liu[1,*], Yu-Chen Zhuang[2,*], Ya-Ning Ren[1,*], Chao Yan[1], Xiao-Feng Zhou[1], Qian Yang[1], Qing-Feng Sun[2,3‡], and Lin He[1‡]

[1]Center for Advanced Quantum Studies, Department of Physics, Beijing Normal University, Beijing 100875, China, and Key Laboratory of Multiscale Spin Physics, Ministry of Education, Beijing 100875, China
[2]International Center for Quantum Materials, School of Physics, Peking University, Beijing 100871, China
[3]Hefei National Laboratory, Hefei 230088, China
[*]These authors contributed equally to this work.
[†]Correspondence and requests for materials should be addressed to Qing-Feng Sun (e-mail: sunqf@pku.edu.cn) and Lin He (e-mail: helin@bnu.edu.cn).

In Supplementary Note 1, we provide a detailed analysis to demonstrate the emergence of two wavefront dislocations in Fast Fourier Transform (FFT)-filtered local density of states (LDOS) without a potential field or under a rotation-symmetric field. Subsequently, in Supplementary Note 2, we reveal the appearance of a single wavefront dislocation in FFT-filtered LDOS under an asymmetric potential by considering the contribution from the scattering between different orbital angular momentum states. The corresponding numerical calculation methods and details are also presented in Supplementary Note 3 and 4.

Supplementary Note 1. Double wavefront dislocations induced by the defect on the pristine graphene

The low-energy properties of the massless relativistic electrons in graphene are well described by the low-energy Hamiltonian of the basis in sublattice space ($|A\rangle,\ |B\rangle$) (ref. [1]):

$$\mathbf{H}(\mathbf{K}_{\boldsymbol{\xi}}+\mathbf{q})=\hbar v_f\begin{pmatrix}0 & \xi q_x-iq_y\\ \xi q_x+iq_y & 0\end{pmatrix}=\hbar v_f\begin{pmatrix}0 & \xi q e^{-i\xi\theta_{\mathbf{q}}}\\ \xi q e^{i\xi\theta_{\mathbf{q}}} & 0\end{pmatrix},\quad (1)$$

where $\xi=\pm$ and $\mathbf{K}_{\pm}$ (or $\mathbf{K}/\mathbf{K}'$) denotes two inequivalent valleys. Here $\mathrm{q}=\sqrt{q_x^2+q_y^2}$ and $\theta_{\mathbf{q}}=arctan\left(\frac{q_y}{q_x}\right)$ is the norm and polar angle of wave vector $\mathbf{q}$ respective to $\mathbf{K}_{\pm}$. The eigenvalues for valley $\mathbf{K}_{\xi}$ are $E_{\pm}^{\xi}(q)=\pm\hbar v_f q$ and the corresponding eigenstates: $\left|u_{\pm}^{\xi}(\mathbf{q})\right\rangle=\frac{1}{\sqrt{2}}\left(|A\rangle\pm\xi e^{i\xi\theta_{\mathbf{q}}}|B\rangle\right)$. The eigenvector defines the pseudospin vectors in the sublattice Bloch space:

$$\left\langle u_{\pm}^{\xi}(\mathbf{q})\middle|\boldsymbol{\sigma}\middle|u_{\pm}^{\xi}(\mathbf{q})\right\rangle=\pm\xi\left(\cos\xi\theta_{\mathbf{q}},\sin\xi\theta_{\mathbf{q}},0\right),\quad (2)$$

which shows the pseudospin-momentum locking texture (as shown in Fig. 1c). Along an anticlockwise closed loop circulating the Dirac point on the Fermi surface, the pseudospin will rotate by 2π to yield a $\mathrm{W}=\xi=$

± 1 winding number as well as $\gamma = \mathrm{i}\oint \left\langle u_{\pm}^{\xi}(\mathbf{q}) \middle| \nabla_q \middle| u_{\pm}^{\xi}(\mathbf{q}) \right\rangle \cdot \mathrm{d}\mathbf{q} = \xi\pi = \mathrm{W}\pi$ Berry phase[1-3].

For each Hamiltonian $\mathbf{H}(\mathbf{K}_{\xi} + \mathbf{q}) \equiv \mathbf{H}_{\xi}(\mathbf{q})$, the bare retarded Green function $\boldsymbol{g}^r(\omega, \mathbf{K}_{\xi} + \mathbf{q}) = \boldsymbol{g}_{\xi}^r(\omega, \mathbf{q}) = \left(\omega + i\eta - \mathbf{H}_{\xi}(\mathbf{q})\right)^{-1}$ with ω the energy of electrons and a tiny energy broadening η can be obtained[4]:

$$\boldsymbol{g}_{\xi}^r(\omega, \mathbf{q}) = \frac{1}{\omega^2 - (\hbar v_f q)^2} \begin{pmatrix} \omega & \xi\hbar v_f q e^{-i\xi\theta_{\mathbf{q}}} \\ \xi\hbar v_f q e^{i\xi\theta_{\mathbf{q}}} & \omega \end{pmatrix}. \quad (3)$$

After an inverse Fourier transformation to the real space:

$$\boldsymbol{g}_{\xi}^r(\omega, \mathbf{r}) = \iint \frac{d^2q}{(2\pi)^2} e^{i\mathbf{q}\cdot\mathbf{r}} \boldsymbol{g}^r(\omega, \mathbf{K}_{\xi} + \mathbf{q}) = \iint \frac{d^2q}{(2\pi)^2} e^{i\mathbf{q}\cdot\mathbf{r}} \boldsymbol{g}_{\xi}^r(\omega, \mathbf{q})$$

$$\approx -\frac{\omega}{(2\hbar v_f)^2} \begin{pmatrix} iH_0\left(\frac{\omega r}{\hbar v_f}\right) & -\xi H_1\left(\frac{\omega r}{\hbar v_f}\right) e^{-i\xi\theta_{\mathbf{r}}} \\ -\xi H_1\left(\frac{\omega r}{\hbar v_f}\right) e^{i\xi\theta_{\mathbf{r}}} & iH_0\left(\frac{\omega r}{\hbar v_f}\right) \end{pmatrix}. \quad (4)$$

Here we have used the integral[5,6]:

$$\iint \frac{d^2q}{(2\pi)^2} \frac{q^{2m} e^{i\mathbf{q}\cdot\mathbf{r}}}{\omega^2 - q^{2l}} \left(q e^{i\xi\theta_{\mathbf{q}}}\right)^n \simeq -\frac{i^{n+1}\omega^{n/l} e^{i\xi n\theta_{\mathbf{r}}}}{4l\omega^{2[1-(m+1)/l]}} H_n\left(\omega^{1/l} r\right), \quad (5)$$

with the integer number *m*, *n* and *l*. H_n denotes the *n*th order Hankel function of the first kind. r and $\theta_{\mathbf{r}}$ is the radius and polar angle relative to the center. In Eqs. (3,4), a relative phase $-\xi\theta_{\mathbf{q}}$ in the momentum space and $-\xi\theta_{\mathbf{r}}$ in the real space always exists between A and B sublattice, which can be also manifested by the pseudospin-momentum texture in Eq. (2) and shown in Fig. 1c in the main text. It originates from the fact that in the massless Dirac equation, electrons hopping from one sublattice to another should experience a gauge-invariant effective flux quantum[4].

To add the effect of scatterings, we describe the potential induced by the (H) defect localized on the sublattice *A* with a delta function at the origin $\mathbf{r} = 0$.

$$V_{adatom}(\mathbf{r}) = \begin{pmatrix} V_0 & 0 \\ 0 & 0 \end{pmatrix} \delta(\mathbf{r}). \quad (6)$$

Using the Dyson equation, the dressed Green's function of the sample which includes the effect of V_{adatom} can be expressed as[4]:

$$\boldsymbol{G}^r(\omega, \mathbf{r}, \mathbf{r}) = \boldsymbol{g}^r(\omega, 0) + \boldsymbol{g}^r(\omega, \mathbf{r}) \boldsymbol{T}(\omega) \boldsymbol{g}^r(\omega, -\mathbf{r}), \quad (7)$$

where $\boldsymbol{T}(\omega) = \begin{pmatrix} t(\omega) & 0 \\ 0 & 0 \end{pmatrix} = \begin{pmatrix} V_0/\left(1 - V_0 g_{AA}^r(\omega, 0)\right) & 0 \\ 0 & 0 \end{pmatrix}$. Here $\boldsymbol{g}^r(\omega, \mathbf{r})$ is the retarded Green's function of the pristine graphene.

Then, the local density of states (LDOS) of the graphene with a *H* defect can be calculated as:

$$\rho(\omega,\mathbf{r}) = -\frac{1}{\pi} Im[\boldsymbol{G}^r(\omega,\mathbf{r},\mathbf{r})]. \quad (8)$$

To obtain the LDOS modulation originated from the intervalley scattering (measured in the experiment from intervalley Fast Fourier transform (FFT) filtering), we first obtain the Fourier components $\rho(\omega, \mathbf{k} = \mathbf{K} + \mathbf{q})$ where $\Delta\mathbf{K} = \mathbf{K}_+ - \mathbf{K}_-$ and **q** is a small number:

$$\rho(\omega, \mathbf{k} = \Delta\mathbf{K} + \mathbf{q}) = \iint d^2r e^{-i(\Delta\mathbf{K}+\mathbf{q})\cdot\mathbf{r}} \rho(\omega,\mathbf{r})$$

$$= -\frac{1}{\pi} \iint d^2r e^{-i(\Delta\mathbf{K}+\mathbf{q})\cdot\mathbf{r}} Im\{Tr[\boldsymbol{G}^r(\omega,\mathbf{r},\mathbf{r})]\}$$

$$\approx \frac{i}{(2\pi)^3} \iint d^2q_1\, Tr[\boldsymbol{g}^r(\omega, \mathbf{K}_+ + \mathbf{q} + \mathbf{q_1})\boldsymbol{T}(\omega)\boldsymbol{g}^r(\omega, \mathbf{K}_- + \mathbf{q_1}) - \boldsymbol{g}^{r,*}(\omega, \mathbf{K}_- - \mathbf{q} + \mathbf{q_1})\boldsymbol{T}^*(\omega)\boldsymbol{g}^{r,*}(\omega, \mathbf{K}_+ + \mathbf{q_1})]$$

$$= \frac{i}{(2\pi)^3} \iint d^2q_1\, Tr[\boldsymbol{g}_+^r(\omega, \mathbf{q} + \mathbf{q_1})\boldsymbol{T}(\omega)\boldsymbol{g}_-^r(\omega, \mathbf{q_1}) - \boldsymbol{g}_-^{r,*}(\omega, -\mathbf{q} + \mathbf{q_1})\, \boldsymbol{T}^*(\omega)\, \boldsymbol{g}_+^{r,*}(\omega, \mathbf{q_1})]. \quad (9)$$

In the FFT in the STM experiment, $\rho(\omega, \mathbf{k} = \Delta\mathbf{K} + \mathbf{q})$ denotes the peaks at the intervalley scattering points $\mathbf{k} = \Delta\mathbf{K}$. Now, we do the filtering by only retaining the values of Fourier components $\rho(\omega, \mathbf{k})$ at around $\mathbf{k} = \Delta\mathbf{K}$ but discarding the other. Then, using the inverse Fourier transform, we integrate these values to obtain FFT-filtered LDOS at $\mathbf{k} = \Delta\mathbf{K}$ for $\rho(\omega, \mathbf{r}, \Delta\mathbf{K})$:

$$\rho(\omega, \mathbf{r}, \Delta\mathbf{K}) = \iint \frac{d^2q}{(2\pi)^2} e^{i(\Delta\mathbf{K}+\mathbf{q})\cdot\mathbf{r}} \rho(\omega, \mathbf{k} = \Delta\mathbf{K} + \mathbf{q})$$

$$= \frac{i}{2\pi} e^{i\Delta\mathbf{K}\cdot\mathbf{r}} Tr[\boldsymbol{g}_+^r(\omega,\mathbf{r})\boldsymbol{T}(\omega)\boldsymbol{g}_-^r(\omega,-\mathbf{r}) - \boldsymbol{g}_-^{r,*}(\omega,\mathbf{r})\boldsymbol{T}^*(\omega)\boldsymbol{g}_+^{r,*}(\omega,-\mathbf{r})]. \quad (10)$$

Thus, the intervalley FFT-filtered LDOS $\Delta\rho(\omega, \mathbf{r})$ measured in the experiment is:

$$\Delta\rho(\omega,\mathbf{r}) = \rho(\omega,\mathbf{r},\Delta\mathbf{K}) + \rho(\omega,\mathbf{r},-\Delta\mathbf{K})$$

$$= -\frac{1}{\pi} Im\{e^{i\Delta\mathbf{K}\cdot\mathbf{r}} Tr[\boldsymbol{g}_+^r(\omega,\mathbf{r})\boldsymbol{T}(\omega)\boldsymbol{g}_-^r(\omega,-\mathbf{r}) - \boldsymbol{g}_-^{r,*}(\omega,\mathbf{r})\boldsymbol{T}^*(\omega)\boldsymbol{g}_+^{r,*}(\omega,-\mathbf{r})]\}, \quad (11)$$

Here $\boldsymbol{g}_\xi^r(\omega,\mathbf{r}) = \iint \frac{d^2q}{(2\pi)^2} e^{i\mathbf{q}\cdot\mathbf{r}} \boldsymbol{g}_\xi^r(\omega,\mathbf{q})$ around the $\mathbf{K}_\xi$ valley and just corresponds to the retarded green's function of Hamiltonian $\boldsymbol{H}_\xi$ in Eq. (4). $\pm\Delta\mathbf{K}$ correspond to filtering points denoted by red circles in Figs. 2c,g. Substituting Eq. (4) into Eq. (11), we could obtain $\Delta\rho(\omega,\mathbf{r}) = \Delta\rho_A(\omega,\mathbf{r}) + \Delta\rho_B(\omega,\mathbf{r})$:

$$\Delta\rho_A(\omega,\mathbf{r}) \approx C Im\left[\omega^2 H_0^2\left(\frac{\omega r}{\hbar v_f}\right) t(\omega)\right] \cos(\Delta\mathbf{K}\cdot\mathbf{r}) = \Delta\tilde{\rho}_A \cos(\Delta\mathbf{K}\cdot\mathbf{r}),$$

$$\Delta\rho_B(\omega,\mathbf{r}) \approx -C Im\left[\omega^2 H_1^2\left(\frac{\omega r}{\hbar v_f}\right) t(\omega)\right] \cos(\Delta\mathbf{K}\cdot\mathbf{r} + 2\theta_\mathbf{r}) = -\Delta\tilde{\rho}_B \cos(\Delta\mathbf{K}\cdot\mathbf{r} + 2\theta_\mathbf{r}), \quad (12)$$

with $C = \frac{1}{8\pi(\hbar v_f)^4}$. In Eq. (12), the total intervalley FFT-filtered LDOS $\Delta\rho(\omega,\mathbf{r})$ is composed of two distinct sublattice contributions $\Delta\rho_A(\omega,\mathbf{r})$ and $\Delta\rho_B(\omega,\mathbf{r})$ which both mainly exhibit the oscillation of $\cos(\Delta\mathbf{K}\cdot\mathbf{r})$ due to the intervalley scatterings. Especially, $\Delta\tilde{\rho}_A(\omega,\mathbf{r})$ and $\Delta\tilde{\rho}_B(\omega,\mathbf{r})$ define the charge modulation induced by the intravalley scattering which describes the Friedel oscillation via $2q_F$ wave vector dependence

(q_F is the Fermi wavevector)[7]. It is worth noting that $\Delta\rho_B(\omega,\mathbf{r})$ involves the scatterings process from B sublattice to A sublattice, and again returns to B sublattice (as shown in Fig. 1a in the main text). The former path and the latter path each carries a phase $\theta_\mathbf{r}$ and then the total path contributes an additional phase $2\theta_\mathbf{r}$ into the $\Delta\rho_B(\omega,\mathbf{r})$. This phase $\Delta\mathbf{K}\cdot\mathbf{r}+2\theta_\mathbf{r}$ of $\Delta\rho_B(\omega,\mathbf{r})$ acts like a potential field whose gradient is a sum of a uniform field and a vortex which is singular at the origin $\mathbf{r}=0$ (ref. [4,8]). Since $\Delta\rho_B(\omega,\mathbf{r})$ is a single-valued function, it must return to the same value after circulating a closed path. Thus, the path circulating around the central defect should contribute a phase 4π as well as 2 additional wavefronts (see Supplementary Figure 1 as an example for $\Delta\rho_B(\omega,\mathbf{r})$). Especially, this 4π is directly related to four times the Berry phase $\gamma=\pi$ and $\mathrm{W}=1$ winding number of the Dirac cone, because the circulation of $\theta_\mathbf{r}$ in the real space is equivalent to the circulation of $\theta_\mathbf{q}=\theta_\mathbf{r}-\pi$ in the momentum space (see Fig. 2a in the main text). While for $\Delta\rho_A(\omega,\mathbf{r})$, it does not carry any phase singularity and contribute wavefront dislocations. Furthermore, the total intervalley FFT-filtered LDOS $\Delta\rho(\omega,\mathbf{r})$ can be also formulated as a real part of a complex scalar field[4]:

$$\Delta\rho(\omega,\mathbf{r})=\mathrm{Re}\left[\Delta\tilde{\rho}_A(\omega,r)e^{i\Delta\mathbf{K}\cdot\mathbf{r}}-\Delta\tilde{\rho}_B(\omega,r)e^{i(\Delta\mathbf{K}\cdot\mathbf{r}+2\theta_\mathbf{r})}\right]=\mathrm{Re}\left[\Delta\tilde{\rho}_{AB}e^{i\varphi_r}\right] \quad (13)$$

where the φ_r is the phase of the complex scalar field:

$$\varphi_r=\Delta\mathbf{K}\cdot\mathbf{r}+\mathrm{Arg}\left[\Delta\tilde{\rho}_A(\omega,r)-\Delta\tilde{\rho}_B(\omega,r)e^{i2\theta_\mathbf{r}}\right]. \quad (14)$$

The phase becomes singular at $\left|\Delta\tilde{\rho}_A(\omega,r)-\Delta\tilde{\rho}_B(\omega,r)e^{i2\theta_\mathbf{r}}\right|=0$. It indicates the position $\mathbf{r}$ where wavefront dislocation could emerge. Due to the characteristics of $2\theta_\mathbf{r}$, the positions of the wavefront dislocation always appear in pairs. Specifically, the positions of the wavefront dislocation satisfy $\Delta\tilde{\rho}_A(\omega,r)=\Delta\tilde{\rho}_B(\omega,r)$ with $\theta_\mathbf{r}=0,\pi$, or satisfy $\Delta\tilde{\rho}_A(\omega,r)=-\Delta\tilde{\rho}_B(\omega,r)$ with $\theta_r=\pm\frac{\pi}{2}$. Totally speaking, for a path circulating the central defect where $|\Delta\tilde{\rho}_A(\omega,r)|<|\Delta\tilde{\rho}_B(\omega,r)|$, there should be two wavefront dislocations within the contour. Usually, two wavefront dislocations near the defect are easily observed and long-distance dislocations are fuzzy. In Fig. 1d in the main text, we schematically show the total charge modulation $\Delta\rho(\omega,r)=\Delta\rho_A(\omega,\mathbf{r})+\Delta\rho_B(\omega,\mathbf{r})$ based on Eq. (12). Ignoring the constant C, we optionally choose $\omega=0.4$ eV, $\hbar v_f\approx 0.73\ eV\cdot nm$ ($v_f\approx 1.1\times10^6\ m/s$), $V_0=50\ eV\cdot nm^2$. Near the defect $\mathrm{r}=0$, the total charge density modulation in Eq. (12) could diverge, where we cut-off it as a white dot[4]. The appearance of double Y-shaped wavefront dislocations in Fig. 1d is also consistent with our former theoretical analysis.

Supplementary Note 2. Single-wavefront dislocations under a broken rotation-symmetry potential field

The derivations in the above note clearly demonstrate two additional wavefronts in intervalley FFT-filtered LDOS $\Delta\rho(\omega,\mathbf{r})$ is attributed to the emergence of phase singularity $\Delta\mathbf{K}\cdot\mathbf{r}+2\theta_\mathbf{r}$, which is attributed to the interference between pseudospin vectors from two distinct valleys, as shown in the Fig. 1c in the main text. Essentially, this contribution corresponds to the topology of the Dirac cone. In this note, we investigate how

an additional broken rotation-symmetry potential field could induce additional phase contribution in Eq. (12) from the orbital angular momentum to modulate the situation of wavefront dislocation. At this time, we consider that there are two potentials acting on graphene: one is a very local potential $V_{adatom}(\mathbf{r})$ induced by the H defect, and the other is a potential $V(\mathbf{r})$ spreading a range around the H defect. The potential $V_{adatom}(\mathbf{r})$ is dependent on the index of the A and B sublattices (see Eq. (6)), but the potential $V(\mathbf{r})$ changes with the coordinate $\mathbf{r} = (x, y)$ and it can be set to be equal at the *A* and *B* sublattices.

We first rephrase the Dirac equation in Eq. (1) to include the potential $V(x, y)$ spreading a range around the defect:

$$\boldsymbol{H}_\xi = \hbar v_f \begin{pmatrix} V(x,y)/\hbar v_f & -i\xi\partial_x - \partial_y \\ -i\xi\partial_x - \partial_y & V(x,y)/\hbar v_f \end{pmatrix}$$

$$= \hbar v_f \begin{pmatrix} V(r,\theta_{\mathbf{r}})/\hbar v_f & -i\xi e^{-i\xi\theta_{\mathbf{r}}}\partial_r - \frac{e^{-i\xi\theta_{\mathbf{r}}}}{r}\partial_{\theta_{\mathbf{r}}} \\ -i\xi e^{i\xi\theta_{\mathbf{r}}}\partial_r + \frac{e^{i\xi\theta_r}}{r}\partial_{\theta_{\mathbf{r}}} & V(r,\theta_{\mathbf{r}})/\hbar v_f \end{pmatrix}. \quad (15)$$

Here, the wave vector $\mathbf{q}$ (q_x, q_y or q, $\theta_{\mathbf{q}}$) in Eq. (1) have been replaced by $-i\boldsymbol{\nabla}$ ($-i\partial_x$, $-i\partial_y$ or $-i\partial_r$, $-i\frac{1}{r}\partial_{\theta_{\mathbf{r}}}$) due to the both rotation symmetry and translation invariance broken by the potential $V(x, y)$. To expediently introduce the orbital angular momentum, we express $\boldsymbol{H}_\xi$ in the polar coordinate $\left(r = \sqrt{x^2+y^2}, \theta_{\mathbf{r}} = \arctan\left(\frac{y}{x}\right)\right)$ where the origin is naturally defined by the defect. Then we expand the eigenstates $\varphi_\xi(r,\theta_{\mathbf{r}}) = \sum_{\widetilde{m}} \frac{e^{i\widetilde{m}\theta_{\mathbf{r}}}}{\sqrt{r}} \begin{pmatrix} \varphi_A^{\widetilde{m}}(r) e^{-i\xi\frac{\theta_{\mathbf{r}}}{2}} \\ i\xi\varphi_B^{\widetilde{m}}(r) e^{i\xi\frac{\theta_{\mathbf{r}}}{2}} \end{pmatrix}$. Here $\widetilde{m} = \pm\frac{1}{2}, \pm\frac{3}{2}, \ldots$ is the half-integer angular quantum number which corresponds to the orbital angular momentum $m = \widetilde{m} - \frac{\xi}{2} = 0, \pm 1, \pm 2, \ldots$ respectively. That is, the $\theta_{\mathbf{r}}$ dependence of $\varphi_\xi(r,\theta_{\mathbf{r}})$ can be divided into two parts: one is the relative phase $\xi\theta_{\mathbf{r}}$ between A and B sublattice from the pseudospin texture (Fig. 1c in the main text), the other is a common phase $m\theta_{\mathbf{r}}$ on both *A* and *B* sublattice from the orbital angular momentum contribution. Through this expansion, we further obtain the eigen-equations:

$$\boldsymbol{H}_\xi \varphi_\xi(r,\theta_{\mathbf{r}}) = \varepsilon \varphi_\xi(r,\theta_{\mathbf{r}}) \Rightarrow \sum_{\widetilde{m}'} \boldsymbol{H}^\xi_{\widetilde{m},\widetilde{m}'} \begin{pmatrix} \varphi_A^{\widetilde{m}'}(r) \\ \varphi_B^{\widetilde{m}'}(r) \end{pmatrix} = \varepsilon \begin{pmatrix} \varphi_A^{\widetilde{m}}(r) \\ \varphi_B^{\widetilde{m}}(r) \end{pmatrix} \quad (16)$$

where $\boldsymbol{H}^\xi_{\widetilde{m},\widetilde{m}'}$ is

$$\boldsymbol{H}^\xi_{\widetilde{m},\widetilde{m}'}(r,\partial_r) = \begin{pmatrix} V_{\widetilde{m},\widetilde{m}'}(r) & 0 \\ 0 & V_{\widetilde{m},\widetilde{m}'}(r) \end{pmatrix} + \hbar v_f \begin{pmatrix} 0 & \partial_r + \xi\frac{\widetilde{m}'}{r} \\ -\partial_r + \xi\frac{\widetilde{m}'}{r} & 0 \end{pmatrix} \delta_{\widetilde{m},\widetilde{m}'} \quad (17)$$

The $V_{\widetilde{m},\widetilde{m}'}(r)=\frac{1}{2\pi}\int_{-\pi}^{\pi}V(r,\theta_{\mathbf{r}})e^{i(\widetilde{m}'-\widetilde{m})\theta_{\mathbf{r}}}d\theta_{\mathbf{r}}$ is the Fourier transform of $V(r,\theta_{\mathbf{r}})$ for the polar angle. The second term in Eq. (17) is the Dirac Hamiltonian in the pristine graphene with the rotational symmetry. Once the potential $V(r,\theta_{\mathbf{r}})$ breaks the rotation symmetry, different orbital angular momentum states should be coupled in view of $V_{\widetilde{m}\neq\widetilde{m}'}(r)\neq 0$ and $\boldsymbol{H}^{\xi}_{\widetilde{m},\widetilde{m}'}(r,\partial_r)$ is not diagonal in the $|\widetilde{m}\rangle$ basis. Notice that we ignore the intervalley scatterings induced by $V(r,\theta_{\mathbf{r}})$ since it is usually so small. Based on the real space Hamiltonian in Eq. (15), we in principle could obtain the real space green's functions $\boldsymbol{g}^r(\omega,\mathbf{r_1},\mathbf{r_2})$ for the graphene with the potential $V(r,\theta_{\mathbf{r}})$. Then by using Dyson equation, we can further get the dressed Green's function of the sample which includes the effect of both the potential $V(r,\theta_{\mathbf{r}})$, and the defect's local potential $V_{adatom}(\mathbf{r})$ in Eq. (6). Even though the translation invariance is lacking in $\boldsymbol{g}^r(\omega,\mathbf{r_1},\mathbf{r_2})$, the dressed Green's function $\boldsymbol{G}^r(\omega,\mathbf{r},\mathbf{r})$ still has a similar form:

$$\boldsymbol{G}^r(\omega,\mathbf{r},\mathbf{r})=\boldsymbol{g}^r(\omega,\mathbf{r},\mathbf{r})+\boldsymbol{g}^r(\omega,\mathbf{r},0)\boldsymbol{T}(\omega)\boldsymbol{g}^r(\omega,0,\mathbf{r}) \quad (18)$$

where $\boldsymbol{T}(\omega)=\begin{pmatrix}t(\omega) & 0\\ 0 & 0\end{pmatrix}=\begin{pmatrix}V_0/\left(1-V_0 g^r_{AA}(\omega,0{,}0)\right) & 0\\ 0 & 0\end{pmatrix}$. Following a similar procedure in Eq. (8) to Eq. (11), the intervalley FFT-filtered LDOS $\Delta\rho(\omega,\mathbf{r})$ still share a form:

$$\begin{aligned}\Delta\rho(\omega,\mathbf{r})&=\rho(\omega,\mathbf{r},\Delta\mathbf{K})+\rho(\omega,\mathbf{r},-\Delta\mathbf{K})\\ &=-\frac{1}{\pi}Im\{e^{i\Delta\mathbf{K}\cdot\mathbf{r}}Tr[\boldsymbol{g}^r_{+}(\omega,\mathbf{r},0)T(\omega)\boldsymbol{g}^r_{-}(\omega,0,\mathbf{r})-\boldsymbol{g}^{r,*}_{-}(\omega,\mathbf{r},0)T^*(\omega)\boldsymbol{g}^{r,*}_{+}(\omega,0,\mathbf{r})]\}. \quad (19)\end{aligned}$$

In this formula, notice that the parameter $\boldsymbol{g}^r_{\xi}(\omega,\mathbf{r_1},\mathbf{r_2})=\iint\iint\frac{d^2q_1d^2q_2}{(2\pi)^4}e^{i\mathbf{q_1}\cdot\mathbf{r_1}-i\mathbf{q_2}\cdot\mathbf{r_2}}\boldsymbol{g}^r_{\xi}(\omega,\mathbf{q_1},\mathbf{q_2})=\iint\iint\frac{d^2q_1d^2q_2}{(2\pi)^4}e^{i\mathbf{q_1}\cdot\mathbf{r_1}-i\mathbf{q_2}\cdot\mathbf{r_2}}\boldsymbol{g}^r\left(\omega,\mathbf{K_\xi}+\mathbf{q_1},\mathbf{K_\xi}+\mathbf{q_2}\right)$ is the double inverse Fourier transform near $\mathbf{K_\xi}$ valley and just corresponds to the Green's functions of $\boldsymbol{H}_{\xi}$ in Eq. (15).

To further analyze the effect of orbital angular momentum on Eq. (19), now we try to construct the relation between the Hamiltonian $\boldsymbol{H}^{\xi}_{\widetilde{m},\widetilde{m}'}(r,\partial_r)$ in Eq. (17) and the retarded Green's function $\boldsymbol{g}^r_{\xi}(\omega,\mathbf{r},\mathbf{r}')$. The retarded Green's function satisfies the equation of motion:

$$(\omega+i\eta)\boldsymbol{g}^r_{\xi}(r,\theta_{\mathbf{r}},r',\theta'_{\mathbf{r}})=\delta(r-r')\delta(\theta_{\mathbf{r}}-\theta'_{\mathbf{r}})+\boldsymbol{H}_{\xi}\left(r,\theta_{\mathbf{r}},\partial_{\mathbf{r}},\partial_{\theta_{\mathbf{r}}}\right)\boldsymbol{g}^r_{\xi}(r,\theta_{\mathbf{r}},r',\theta'_{\mathbf{r}}). \quad (20)$$

Then we multiply $\sqrt{r}\boldsymbol{U}^{\dagger}_{\xi}(\theta_{\mathbf{r}})$ on the left and $\boldsymbol{U}_{\xi}(\theta'_{\mathbf{r}})$ on the right where $\boldsymbol{U}_{\xi}(\theta_{\mathbf{r}})=\begin{pmatrix}e^{-i\xi\frac{\theta_{\mathbf{r}}}{2}} & 0\\ 0 & i\xi e^{i\xi\frac{\theta_{\mathbf{r}}}{2}}\end{pmatrix}$ to do the unitary transformation and obtain:

$$\begin{aligned}(\omega+i\eta)\sqrt{r}\widetilde{\boldsymbol{g}}^r_{\xi}(r,\theta_{\mathbf{r}},r',\theta'_{\mathbf{r}})&=\sqrt{r}\boldsymbol{U}^{\dagger}_{\xi}(\theta_r)\delta(r-r')\delta(\theta_{\mathbf{r}}-\theta'_{\mathbf{r}})\boldsymbol{U}_{\xi}(\theta'_{\mathbf{r}})\\ &+\mathrm{r}\frac{\boldsymbol{U}^{\dagger}_{\xi}(\theta_{\mathbf{r}})}{\sqrt{r}}\boldsymbol{H}_{\xi}\left(r,\theta_{\mathbf{r}},\partial_r,\partial_{\theta_{\mathbf{r}}}\right)\frac{\boldsymbol{U}_{\xi}(\theta_{\mathbf{r}})}{\sqrt{r}}\sqrt{r}\widetilde{\boldsymbol{g}}^r_{\xi}(r,\theta_{\mathbf{r}},r',\theta'_{\mathbf{r}}) \quad (21)\end{aligned}$$

Where we define $\tilde{\boldsymbol{g}}_{\xi}^{r}(r,\theta_{\mathbf{r}},r',\theta_{\mathbf{r}}') \equiv \boldsymbol{U}_{\xi}^{\dagger}(\theta_{\mathbf{r}})\boldsymbol{g}_{\xi}^{r}(r,\theta_{\mathbf{r}},r',\theta_{\mathbf{r}}')\,\boldsymbol{U}_{\xi}(\theta_{\mathbf{r}}')$. Expand it into the basis of $|\tilde{m}\rangle$:

$$\tilde{\boldsymbol{g}}_{\xi}^{r}(r,\theta_{\mathbf{r}},r',\theta_{\mathbf{r}}') = \sum_{\tilde{m}_1,\tilde{m}_2} e^{i\tilde{m}_1\theta_{\mathbf{r}}-i\tilde{m}_2\theta_{\mathbf{r}}'}\,\tilde{\boldsymbol{g}}_{\tilde{m}_1\tilde{m}_2}^{r,\xi}(r,r'). \quad (22)$$

Put Eq. (22) into Eq. (21), multiply $e^{-i\tilde{m}\theta_{\mathbf{r}}}/e^{i\tilde{n}\theta_{\mathbf{r}}'}$ on the left/right side of Eq. (21) respectively, integrate $\theta_{\mathbf{r}}$ and $\theta_{\mathbf{r}}'$ over the range of 2π, the Eq. (21) can be finally reformulated as:

$$(\omega+i\eta)\sqrt{r}\tilde{\boldsymbol{g}}_{\tilde{m}\tilde{n}}^{r,\xi}(r,r') = \sqrt{r}\delta(r-r')\,\delta_{\tilde{m}\tilde{n}} + \sum_{\tilde{m}_1}\boldsymbol{H}_{\tilde{m}\tilde{m}_1}^{\xi}(r,\partial_r)\sqrt{r}\tilde{\boldsymbol{g}}_{\tilde{m}_1\tilde{n}}^{r,\xi}(r,r'). \quad (23)$$

Here $\boldsymbol{H}_{\tilde{m}\tilde{n}}^{\xi}(r,\partial_r)$ is just consistent with Hamiltonian in Eq. (17). In principle, as long as we know $\tilde{\boldsymbol{g}}_{\tilde{m}\tilde{n}}^{r,\xi}(r,r')$ the solution of Eq. (23), $\boldsymbol{g}_{\xi}^{r}(r,\theta_{\mathbf{r}},r',\theta_{\mathbf{r}}')$ as well as $\Delta\rho(\omega,\mathbf{r})$ in Eq. (19) can be obtained straightforwardly. In detail, $\boldsymbol{g}_{\xi}^{r}(r,\theta_{\mathbf{r}},r',\theta_{\mathbf{r}}')$ satisfies the form:

$$\boldsymbol{g}_{\xi}^{r}(r,\theta_{\mathbf{r}},r',\theta_{\mathbf{r}}') = \sum_{\tilde{m}_1,\tilde{m}_2} e^{i\tilde{m}_1\theta_{\mathbf{r}}-i\tilde{m}_2\theta_{\mathbf{r}}'}\,\boldsymbol{U}_{\xi}(\theta_{\mathbf{r}})\tilde{\boldsymbol{g}}_{\tilde{m}_1\tilde{m}_2}^{r,\xi}(r,r')\boldsymbol{U}_{\xi}^{\dagger}(\theta_{\mathbf{r}}')$$

$$= \sum_{\tilde{m}_1\tilde{m}_2}\begin{pmatrix} \tilde{g}_{AA;\tilde{m}_1\tilde{m}_2}^{r,\xi}(r,r')e^{i\left(\tilde{m}_1-\frac{\xi}{2}\right)\theta_{\mathbf{r}}-i\left(\tilde{m}_2-\frac{\xi}{2}\right)\theta_{\mathbf{r}}'} & -i\xi\tilde{g}_{AB;\tilde{m}_1\tilde{m}_2}^{r,\xi}(r,r')e^{i\left(\tilde{m}_1-\frac{\xi}{2}\right)\theta_{\mathbf{r}}-i\left(\tilde{m}_2+\frac{\xi}{2}\right)\theta_{\mathbf{r}}'} \\ i\xi\tilde{g}_{BA;\tilde{m}_1\tilde{m}_2}^{r,\xi}(r,r')e^{i\left(\tilde{m}_1+\frac{\xi}{2}\right)\theta_{\mathbf{r}}-i\left(\tilde{m}_2-\frac{\xi}{2}\right)\theta_{\mathbf{r}}'} & \tilde{g}_{BB;\tilde{m}_1\tilde{m}_2}^{r,\xi}(r,r')e^{i\left(\tilde{m}_1+\frac{\xi}{2}\right)\theta_{\mathbf{r}}-i\left(\tilde{m}_2+\frac{\xi}{2}\right)\theta_{\mathbf{r}}'} \end{pmatrix}. \quad (24)$$

Based on the Eq. (24), the corresponding $\boldsymbol{g}_{\xi}^{r}(r,\theta_{\mathbf{r}},r'=0)$, $\boldsymbol{g}_{\xi}^{r}(r=0,r',\theta_{\mathbf{r}}')$, and $\boldsymbol{g}_{\xi}^{r}(r=0,r'=0)$ can be evaluated as:

$$\boldsymbol{g}_{\xi}^{r}(r,\theta_{\mathbf{r}},r'=0) = \int_{-\pi}^{\pi}\frac{d\theta_{\mathbf{r}}'}{2\pi}\boldsymbol{g}_{\xi}^{r}(r,\theta_{\mathbf{r}},r'=0,\theta_{\mathbf{r}}') =$$

$$\sum_{\tilde{m}_1}\begin{pmatrix} \tilde{g}_{AA;\tilde{m}_1\frac{\xi}{2}}^{r,\xi}(r,0)e^{i\left(\tilde{m}_1-\frac{\xi}{2}\right)\theta_{\mathbf{r}}} & -i\xi\tilde{g}_{AB;\tilde{m}_1,\frac{-\xi}{2}}^{r,\xi}(r,0)e^{i\left(\tilde{m}_1-\frac{\xi}{2}\right)\theta_{\mathbf{r}}} \\ i\xi\tilde{g}_{BA;\tilde{m}_1\frac{\xi}{2}}^{r,\xi}(r,0)e^{i\left(\tilde{m}_1+\frac{\xi}{2}\right)\theta_{\mathbf{r}}} & \tilde{g}_{BB;\tilde{m}_1\frac{-\xi}{2}}^{r,\xi}(r,0)e^{i\left(\tilde{m}_1+\frac{\xi}{2}\right)\theta_{\mathbf{r}}} \end{pmatrix},$$

$$\boldsymbol{g}_{\xi}^{r}(r=0,r',\theta_{\mathbf{r}}') = \int_{-\pi}^{\pi}\frac{d\theta_{\mathbf{r}}}{2\pi}\boldsymbol{g}_{\xi}^{r}(r=0,r',\theta_{\mathbf{r}}')$$

$$= \sum_{\tilde{m}_2}\begin{pmatrix} \tilde{g}_{AA;\frac{\xi}{2}\tilde{m}_2}^{r,\xi}(0,r')e^{-i\left(\tilde{m}_2-\frac{\xi}{2}\right)\theta_{\mathbf{r}}'} & -i\xi\tilde{g}_{AB;\frac{\xi}{2}\tilde{m}_2}^{r,\xi}(0,r')e^{-i\left(\tilde{m}_2+\frac{\xi}{2}\right)\theta_{\mathbf{r}}'} \\ i\xi\tilde{g}_{BA;\frac{-\xi}{2}\tilde{m}_2}^{r,\xi}(0,r')e^{-i\left(\tilde{m}_2-\frac{\xi}{2}\right)\theta_{\mathbf{r}}'} & \tilde{g}_{BB;\frac{-\xi}{2}\tilde{m}_2}^{r,\xi}(0,r')e^{-i\left(\tilde{m}_2+\frac{\xi}{2}\right)\theta_{\mathbf{r}}'} \end{pmatrix},$$

$$\boldsymbol{g}_{\xi}^{r}(r=0,r'=0) = \iint\frac{d\theta_{\mathbf{r}}d\theta_{\mathbf{r}}'}{(2\pi)^2}\boldsymbol{g}_{\xi}^{r}(r=0,\theta_{\mathbf{r}},r'=0,\theta_{\mathbf{r}}') = \begin{pmatrix} \tilde{g}_{AA;\frac{\xi}{2}\frac{\xi}{2}}^{r,\xi}(0,0) & -i\xi\tilde{g}_{AB;\frac{\xi}{2},\frac{-\xi}{2}}^{r,\xi}(0,0) \\ i\xi\tilde{g}_{BA,\frac{-\xi}{2}\frac{\xi}{2}}^{r,\xi}(0,0) & \tilde{g}_{BB;\frac{-\xi}{2},\frac{-\xi}{2}}^{r,\xi}(0,0) \end{pmatrix}. \quad (25)$$

Here we integrate the polar angle to mop the uncertainty of $\theta_{\mathbf{r}}$ at $r=0$. From Eq. (19), we can know the intervalley FFT-filtered LDOS $\Delta\rho(\omega,\mathbf{r})$ is:

$$\Delta\rho(\omega,\mathbf{r})=\Delta\rho_A(\omega,\mathbf{r})+\Delta\rho_B(\omega,\mathbf{r})=-\frac{1}{\pi}Im\{e^{i\Delta\mathbf{K}\cdot\mathbf{r}}[a(r,\theta_{\mathbf{r}})+b(r,\theta_{\mathbf{r}})e^{i2\theta_{\mathbf{r}}}]\}$$

$$\Delta\rho_A(\omega,\mathbf{r})=-\frac{1}{\pi}Im\Big\{e^{i\Delta\mathbf{K}\cdot\mathbf{r}}\Big[t(\omega)\sum_{\tilde{m}_1\tilde{m}_2}\tilde{g}^{r,+}_{AA;\tilde{m}_1\frac{1}{2}}(r,0)\tilde{g}^{r,-}_{AA;\frac{-1}{2}\tilde{m}_2}(0,r)e^{i(\tilde{m}_1-\tilde{m}_2-1)\theta_{\mathrm{r}}}-$$

$$t^*(\omega)\sum_{\tilde{m}_1\tilde{m}_2}\tilde{g}^{r*,-}_{AA;\tilde{m}_2\frac{-1}{2}}(r,0)\tilde{g}^{r*,+}_{AA;\frac{1}{2}\tilde{m}_1}(0,r)e^{i(\tilde{m}_1-\tilde{m}_2-1)\theta_{\mathrm{r}}}\Big]\Big\}\equiv-\frac{1}{\pi}Im\{e^{i\Delta\mathbf{K}\cdot\mathbf{r}}a(r,\theta_{\mathbf{r}})\}.\quad(26A)$$

$$\Delta\rho_B(\omega,\mathbf{r})=-\frac{1}{\pi}Im\Big\{e^{i(\Delta\mathbf{K}\cdot\mathbf{r}+2\theta_{\mathrm{r}})}\Big[-t(\omega)\sum_{\tilde{m}_3\tilde{m}_4}\tilde{g}^{r,+}_{BA;\tilde{m}_3\frac{1}{2}}(r,0)\tilde{g}^{r,-}_{AB;\frac{-1}{2}\tilde{m}_4}(0,r)e^{i(\tilde{m}_3-\tilde{m}_4-1)\theta_{\mathrm{r}}}+$$

$$t^*(\omega)\sum_{\tilde{m}_3\tilde{m}_4}\tilde{g}^{r*,-}_{BA;\tilde{m}_4\frac{-1}{2}}(r,0)\tilde{g}^{r*,+}_{AB;\frac{1}{2}\tilde{m}_3}(0,r)e^{i(\tilde{m}_3-\tilde{m}_4-1)\theta_{\mathrm{r}}}\Big]\Big\}\equiv-\frac{1}{\pi}Im\{e^{i(\Delta\mathbf{K}\cdot\mathbf{r}+2\theta_{\mathrm{r}})}b(r,\theta_{\mathbf{r}})\}.\quad(26B)$$

Here $t(\omega)=V_0\left(1-V_0\sum_\xi g^{r,\xi}_{AA;\frac{\xi\xi}{22}}(\omega,0,0)\right)^{-1}$. If $V(r,\theta_{\mathbf{r}})$ does not break the rotational symmetry of the system, $\boldsymbol{H}^{\xi}_{\tilde{m}\tilde{n}}(r,\partial_r)$ as well as $\tilde{\boldsymbol{g}}^{r,\xi}_{\tilde{m}_1\tilde{m}_2}(r,r')$ must be diagonal in the half-integer quantum number basis, and thus Eq. (26) can be simplified as:

$$\Delta\rho_A(\omega,\mathbf{r})=-\frac{1}{\pi}Im\left\{e^{i\Delta\mathbf{K}\cdot\mathbf{r}}\left[t(\omega)\tilde{g}^{r,+}_{AA;\frac{11}{22}}(r,0)\tilde{g}^{r,-}_{AA;\frac{-1-1}{2\ 2}}(0,r)-t^*(\omega)\tilde{g}^{r*,-}_{AA;\frac{-1-1}{2\ 2}}(r,0)\tilde{g}^{r*,+}_{AA;\frac{11}{22}}(0,r)\right]\right\}$$

$$\Delta\rho_B(\omega,\mathbf{r})=-\frac{1}{\pi}Im\left\{e^{i(\Delta\mathbf{K}\cdot\mathbf{r}+2\theta_{\mathrm{r}})}\left[-t(\omega)\tilde{g}^{r,+}_{BA;\frac{11}{22}}(r,0)\tilde{g}^{r,-}_{AB;\frac{-1-1}{2\ 2}}(0,r)+t^*(\omega)\tilde{g}^{r*,-}_{BA;\frac{-1-1}{2\ 2}}(r,0)\tilde{g}^{r*,+}_{AB;\frac{11}{22}}(0,r)\right]\right\}.$$

(27)

The Eq. (27) can be regarded as the scattering process for the orbital angular momentum between $\tilde{m}=\frac{1}{2}$ ($m=0$) in $\mathbf{K}$ valley and $\tilde{m}=-\frac{1}{2}$ ($m=0$) in $\mathbf{K}'$ valley. Naturally, an additional $2\theta_{\mathbf{r}}$ phase appears in the intervalley FFT-filtered LDOS $\Delta\rho_B(\omega,\mathbf{r})$ of the B sublattice which is well-consistent with the result in Eq. (13). For a broken rotation symmetry potential $V(\mathbf{r})$, different orbital momentum m will be coupled and induce additional phases both in $\Delta\rho_A(\omega,\mathbf{r})$ and $\Delta\rho_B(\omega,\mathbf{r})$. Commonly, the main contribution comes from the scatterings between the low orbital angular momentum. For example, in the case of $\Delta\rho_B(\omega,\mathbf{r})$ in Eq. (26A) where $m=0$ ($\tilde{m}_3=\frac{1}{2}$) state in $\mathbf{K}$ valley is scattered to $m=1$ ($\tilde{m}_4=\frac{1}{2}$) state in $\mathbf{K}'$ valley (as shown in Fig. 1e), $\Delta\rho_B(\omega,\boldsymbol{r})$ should reduce into ($\pm\Delta\mathbf{K}$ are both considered):

$$\Delta\rho_B(\omega,\mathbf{r})=-\frac{1}{\pi}Im\left\{e^{i(\Delta\mathbf{K}\cdot\mathbf{r}+\theta_{\mathrm{r}})}\left[-t(\omega)\tilde{g}^{r,+}_{BA;\frac{11}{22}}(r,0)\tilde{g}^{r,-}_{AB;\frac{-11}{2\ 2}}(0,r)+t^*(\omega)\tilde{g}^{r*,-}_{BA;\frac{1-1}{2\ 2}}(r,0)\tilde{g}^{r*,+}_{AB;\frac{11}{22}}(0,r)\right]\right\}.\quad(28)$$

Similarly, in the case of $\Delta\rho_A(\omega,\mathbf{r})$ in Eq. (26B) where $m=1$ ($\tilde{m}_1=\frac{3}{2}$) state in $\mathbf{K}$ valley is scattered to $m=0$ ($\tilde{m}_2=-\frac{1}{2}$) state in $\mathbf{K}'$ valley, $\Delta\rho_A(\omega,\mathbf{r})$ should change into:

$$\Delta\rho_A(\omega,\mathbf{r}) = -\frac{1}{\pi} Im\left\{e^{i(\Delta\mathbf{K}\cdot\mathbf{r}+\theta_\mathbf{r})}\left[t(\omega)\tilde{g}^{r,+}_{AA;\frac{3}{2}\frac{1}{2}}(r,0)\tilde{g}^{r,-}_{AA;\frac{-1}{2}\frac{-1}{2}}(0,r) - t^*(\omega)\tilde{g}^{r*,-}_{AA;\frac{-1}{2}\frac{-1}{2}}(r,0)\tilde{g}^{r*,+}_{AA;\frac{1}{2}\frac{3}{2}}(0,r)\right]\right\}. \quad (29)$$

Here note that the FFT-filtered $\Delta\rho_B(\omega,\mathbf{r})$ and $\Delta\rho_A(\omega,\mathbf{r})$ include both $\pm\Delta\mathbf{K}$ intervalley scattering process. In both cases, their phases change into $\Delta\mathbf{K}\cdot\mathbf{r}+\theta_\mathbf{r}$ and thus reflects only one similar additional wavefront in the map of $\Delta\rho_B(\omega,\mathbf{r})$ and $\Delta\rho_A(\omega,\mathbf{r})$.

Overall, the contribution of the orbital angular momentum into $\Delta\rho(\omega,\mathbf{r})$ can be summarized as:

$$\Delta\rho(\omega,\mathbf{r}) = -\frac{1}{\pi} Im\{e^{i\Delta\mathbf{K}\cdot\mathbf{r}}[a(r,\theta_\mathbf{r}) + b(r,\theta_\mathbf{r})e^{i2\theta_\mathbf{r}}]\}$$

$$= -\frac{1}{\pi} Im\{e^{i\Delta\mathbf{K}\cdot\mathbf{r}}\textstyle\sum_{\Delta m}[a(r,\Delta m)e^{i\Delta m\theta_\mathbf{r}} + b(r,\Delta m)e^{i\Delta m\theta_\mathbf{r}}e^{i2\theta_\mathbf{r}}]\}. \quad (30)$$

Here Δm denotes the difference of the orbital angular momentum during the intervalley scattering process. $a(r,\Delta m)$ and $b(r,\Delta m)$ denote the amplitude for scattering process with Δm the change of the orbital angular momentum, which are both determined by the Green's functions in Eq. (26). Most importantly, like the pseudospin, a finite Δm also introduces a phase $\Delta m\theta_\mathbf{r}$. We pay attention on some terms in Eq. (30) which could shift the phase to $\theta_\mathbf{r}$ rather than $2\theta_\mathbf{r}$,

$$\Delta\rho(\omega,\vec{r}) = -\frac{1}{\pi} Im\{e^{i(\Delta\mathbf{K}\cdot\mathbf{r}+\theta_\mathbf{r})}[a(r,\Delta m=1) + b(r,\Delta m=-1)]\} + \Delta\hat{\rho}(\omega,\mathbf{r}). \quad (31)$$

Here $\Delta\hat{\rho}(\omega,\mathbf{r})$ denotes the remaining terms in Eq. (30). In principle, we could expect $a(r,\Delta m=1)$ and $b(r,\Delta m=-1)$ become the main contribution in Eq. (31) near $\mathbf{r}=0$ under a propriate potential $V(r,\theta_\mathbf{r})$ spreading a range around the defect. They approximately contribute a LDOS modulation like $\Delta\rho(\omega,\mathbf{r}) \propto \cos(\Delta\mathbf{K}\cdot\mathbf{r}+\theta_\mathbf{r})$ which only exhibits one additional dislocation near the defect (see an example in Fig. 1f in the main text). In principle, the other terms like $a(r,\Delta m=-1)$ and $b(r,\Delta m=1)$ should also contribute to the LDOS modulation in Eq. (31) and affect the situation of wavefront dislocations. However, this contribution highly relies on the specific distributions $a(r,\Delta m)$ and $b(r,\Delta m)$ and generally induce additional wavefronts away from the defect. To study the emergence of the single wavefront dislocation in depth, we conduct a numerical calculation to include all Δm contributions in the following.

Supplementary Note 3. Parameters setting and details for numerical simulations

To clearly prove our previous analysis, we conduct numerical calculations based on low energy Hamiltonian in Eq. (15). By dividing $\frac{\hbar v_f}{r_*}$, we make Eq. (15) dimensionless with the length unit r_* and energy unit $\varepsilon_* = \frac{\hbar v_f}{r_*}$. Using a finite difference method[3,10], we discretize the radial r position into N sites with $r \in [0, \mathrm{L}]$, and use the single step difference to substitute the derivative ∂_r. Then we obtain a $2N * 2N$ matrix $\boldsymbol{H}^{\xi}_{\widetilde{m},\widetilde{m}'}(i,j)$ for determined half-integer quantum number $\widetilde{m},\widetilde{m}'$ on the site basis $|i\rangle$. Next, we arrange $\boldsymbol{H}^{\xi}_{\widetilde{m},\widetilde{m}'}(i,j)$ to obtain the total $4NM \times 4NM$ Hamiltonian $\boldsymbol{H}^{\xi}$ in the direct product space of $|i\rangle \otimes |\widetilde{m}\rangle$. Here $2M$

denotes the number of truncated half-integer quantum number $\widetilde{m} \in \left[-\frac{2M-1}{2}, \frac{2M-1}{2}\right]$. Based on this finite difference matrix $\boldsymbol{H}^{\xi}$, the corresponding $\widetilde{\boldsymbol{g}}^{r,\xi}_{\widetilde{m}\widetilde{n}}(i,j)$ can be naturally solved from Eq. (23):

$$\widetilde{\boldsymbol{g}}^{r,\xi} = \left((\omega + i\eta)\boldsymbol{I} - \boldsymbol{R}^{-1}\boldsymbol{H}^{\xi}\boldsymbol{R}\right)^{-1} \quad (32)$$

where $\boldsymbol{I}$ is $4NM \times 4NM$ unit matrix in the direct product space of $|i\rangle \otimes |\widetilde{m}\rangle$ and $\boldsymbol{R} = \sqrt{i}\delta_{ij}\delta_{\widetilde{m}\widetilde{m}'}$. Putting the corresponding numerically calculated $\widetilde{\boldsymbol{g}}^{r,\xi}$ into Eq. (26), the $\Delta\rho(\omega, r_k, \theta_l)$ for determined r_k and θ_l can be evaluated. Especially, we introduce a space broadening λ_s to transform $\Delta\rho(\omega, r_k, \theta_l)$ into the $\Delta\rho(\omega, x_i, y_j)$ in the system of rectangular coordinates:

$$\Delta\rho(\omega, x_i, y_j) = \sum_{k,l} \Delta\rho(\omega, r_k, \theta_l)\, e^{-\frac{[x_i - r_k cos(\theta_l)]^2 + [y_j - r_k sin(\theta_l)]^2}{\lambda_s^2}} \quad (33)$$

In detail of the parameters, we set the Fermi velocity $v_f = 1.1 \times 10^6\ m/s$ with $\hbar v_f \approx 0.73\ eV \cdot nm$, $r^* = 10\ nm$ and $\varepsilon_* = \frac{\hbar v_f}{r_*} \approx 0.073\ eV \cdot nm$. The length of $L = 20$ and the range of $\widetilde{m}$ is $M = 10$ which are sufficiently large to make the results converged. The space broadening is $\lambda_s = 0.01$ and energy broadening $\eta = 0.1$. Considering the amplitude of delta potential V_0 for the defect is relatively large[4], we set $V_0 = 30$. In the calculations, we mainly concentrate on the wavefronts within the range around the defect (3.6 nm $\times$ 3.6 nm) as the experiment did and ignore the long-distance oscillations, since the amplitude of these oscillations are relatively small and easily disturbed by the environment.

Supplementary Note 4. Numerical simulation results for double and single-wavefront dislocations

In Fig. 1d in the main text, we plot the numerically calculated intervalley FFT-filtered $\Delta\rho(\omega, \mathbf{r})$ when the potential $V(r, \theta_{\mathbf{r}})$ in Eq. (15) is absent. At this time, the case should be consistent with Supplementary Note 1 and two wavefront dislocations should appear. We set $\Delta\mathbf{K} = \left(-\frac{4\pi}{3\sqrt{3}a_{cc}}, 0\right)$ in Eq. (26) where $a_{cc} = 0.142$ nm is length of the carbon-carbon bond in graphene. In the Fig. 2e, we concentrate on the distribution of $\Delta\rho(\omega, \mathbf{r})$ around the origin where the defect locates and set $\omega =$ 6 (about 0.44 eV). The corresponding curves of $\Delta\tilde{\rho}_A(\omega, r) \propto Im[\omega^2 H_0^2(\omega r)t(\omega)]$ and $\Delta\tilde{\rho}_B(\omega, r) \propto Im[\omega^2 H_1^2(\omega r)t(\omega)]$ with $t(\omega) = V_0/\left(1 - V_0 g_{AA}^r(\omega, 0)\right) \propto 1/\left[V_0^{-1} + \frac{i}{4}\omega H_0(\omega r \longrightarrow 0)\right]$ can be directly calculated by Eq. (4). They are also shown in Supplementary Figure 8a respectively (here r has been converted to nm by multiplying the length unit $r^* =$ 10 nm). In Fig. 2e, the $\Delta\rho(\omega, \mathbf{r})$ exhibits oscillation stripes which the spacing between the stripes is about $\frac{2\pi}{|\Delta\mathbf{K}|} \approx$ 3.7 Å. Two obvious additional wavefronts are symmetrically distributed at both sides of the defect, in which the orientations of their positions are collinear to $\Delta\mathbf{K}$ ($\theta_{\mathbf{r}} = 0, \pi$). They just reflect the situation where $\Delta\tilde{\rho}_A(\omega, r) = \Delta\tilde{\rho}_B(\omega, r)$ (r ≈ 1 nm denoted by the dark circle in Supplementary Figure 8a)

and indicate the phase of $\Delta\rho(\omega,\mathbf{r})$ should additionally accumulate 4π phase after a small circulation of the origin. In principle, more wavefront dislocations should also emerge in pairs, like those perpendicular to $\Delta\mathbf{K}$ ($\theta_{\mathbf{r}}=\pm\frac{\pi}{2}$) where $\Delta\tilde{\rho}_A(\omega,r)=-\Delta\tilde{\rho}_B(\omega,r)$. But these wavefront dislocations are located far away from the origin, thus are not the focus of the experiment and theory.

Next, we consider a rotationally symmetric potential $V(r,\theta_{\mathbf{r}})$ in Eq. (15) and its effect on wavefront dislocation. The numerically calculated intervalley FFT-filtered $\Delta\rho(\omega,\mathbf{r})$ with $\Delta\mathbf{K}=\left(-\frac{4\pi}{3\sqrt{3}a_{cc}},0\right)$ is plotted in Supplementary Figure 8b. Here $V(r,\theta_{\mathbf{r}})$ is set as a Gaussian form $V(r,\theta_{\mathbf{r}})=-V_0e^{-r^2/R^2}$ which is independent of the angle $\theta_{\mathbf{r}}$, where $V_0=3.4$ ($\approx$ 0.25 eV) and $R=$ 1.8 (18 nm). This potential profile basically follows the LDOS distribution in the experiment. Although the introduction of $V(r)$ affects the Dirac equation and the position of Fermi level, it does not additionally alter the phase $\Delta\rho(\omega,\mathbf{r})$. The phase appears in the $\Delta\rho_B(\omega,\mathbf{r})$ in Eq. (30) still shows a characteristic of $2\theta_{\mathbf{r}}$, which is only originated from the distinct pseudospin texture between $\mathbf{K}$ and $\mathbf{K}'$ valley. No inter-orbital angular momentum scatterings can contribute to the Eq. (30) because the orbital angular momentum m is a good quantum number. Thus, in Supplementary Figure 8b with $\omega=2$ ($\approx$ 0.15 eV), two wavefront dislocations symmetrically near the defect are still observed.

Our theoretical analysis indicates that the phenomenon where only one wavefront dislocation appears near the defect could be related to a potential field $V(r,\theta_{\mathbf{r}})$ with a broken rotational symmetry. In this case, orbital angular momentum is no longer a good quantum number, and states of adjacent orbital angular momentum may be coupled to additionally contribute the phase related to $\theta_{\mathbf{r}}$ in the $\Delta\rho(\omega,\mathbf{r})$. In the experiment, such a potential is speculated to be induced by the STM tip and is hard to decide precisely. However, to qualitatively illustrate the single wavefront dislocation observed in experiment results, we directly consider a simple way to add one potential field $V_1(r,\theta_{\mathbf{r}})$ on the Gaussian potential $-V_0e^{-r^2/R^2}$. That is, the potential in Eq. (15) is set as $V(r,\theta_{\mathbf{r}})=-V_0e^{-\frac{r^2}{R^2}}+V_1(r,\theta_{\mathbf{r}})$ with

$$V_1(r,\theta_{\mathbf{r}})=\begin{cases}\bar{V}_1 & \theta_{\mathbf{r}}\in\left[-\frac{\pi}{4},\frac{\pi}{4}\right]\text{ and } r\in(0,r_0]\\ 0 & \text{the other case}\end{cases}\quad(34)$$

Here $V_1(r,\theta_{\mathbf{r}})$ is a step function which only exists in one area as a constant near the defect. To strongly break the rotational symmetry, we set a relatively large potential $\bar{V}_1=$ 10 ($\approx$ 0.73 eV) and $r_0=$ 3 (30 nm) as an example. In fact, some other kinds of $V_1(r,\theta_{\mathbf{r}})$ may also induce a similar change of wavefront dislocation. In numerical calculations, since the expansion of $V_1(r,\theta_{\mathbf{r}})$ involves infinite orders in orbital angular momentum basis, for convenience we expand $V_1(r,\theta_{\mathbf{r}})$ in orbital angular momentum basis with $V^1_{\widetilde{m}-\widetilde{m}'}(r)=\frac{1}{2\pi}\int_{-\pi}^{\pi}V_1(r,\theta_{\mathbf{r}})e^{i(\widetilde{m}'-\widetilde{m})\theta_{\mathbf{r}}}d\theta_{\mathbf{r}}$ into only the first five orders We retain the first five orders $V^1_0(r)$, $V^1_{\pm1}(r)$,…, $V^1_{\pm5}(r)$. Even though this expansion makes $V_1(r,\theta_{\mathbf{r}})$ more extensive than Eq. (34), it is still enough to break rotation symmetry and induce inter-orbital angular momentum coupling. In Fig. 2i in the main text, we show the numerically calculated $\Delta\rho(\omega,\mathbf{r})$ for $\omega=$ 3.5 ($\approx$ 0.26 eV) with $\Delta\mathbf{K}=$

$\left(-\frac{4\pi}{3\sqrt{3}a_{cc}}, 0\right)$. Different from the results shown in Fig. 2e and Supplementary Figure 8, only a single wavefront dislocation is observed in the vicinity of the origin. The main features in Fig. 2i are similar to the experimental results. The discrepancy on the energy is probably caused by the deviation between the simulated potential between the experiment potential. Furthermore, in both experimental and simulation results (see Supplementary Figure 9), we find such a single wavefront dislocation can persist over a range of energy $\omega =$ 2.5, 3, 4 (0.183 eV, 0.22 eV and 0.292 eV), which reflects the main contribution of $\Delta\mathbf{K} \cdot \mathbf{r} + \theta_{\mathbf{r}}$ in Eq. (31) is robust. Of course, too high or too low electron energy ω may weaken the effect of $V_1(r, \theta_{\mathbf{r}})$, reduce the probability of scatterings between orbital angular momentums and thus make the contribution of phase $2\theta_{\mathbf{r}}$ in $\Delta\rho(\omega, \mathbf{r})$ dominates again.

In Supplementary Figure 10a,b, we also investigate the $\Delta\rho(\omega, \mathbf{r})$ under the potential field $V(r, \theta_{\mathbf{r}}) = -V_0 e^{-\frac{r^2}{R^2}} + V_1(r, \theta_{\mathbf{r}})$ for the other two intervalley FFT-filtering directions with $\omega = 2$ (≈ 0.15 eV) and $\Delta\mathbf{K} = \left(\frac{2\pi}{3\sqrt{3}a_{cc}}, \mp\frac{2\pi}{3a_{cc}}\right)$. Similar to Supplementary Figure 9a, the direction of wavefront fringes exhibits 120 degrees and 240 degrees rotating respectively in Supplementary Figure 10a,b, which is just perpendicular to $\Delta\mathbf{K}$. Clearly, the change of intervalley FFT-filtering direction does not affect the situations of the single wavefront dislocation. This can be directly understood from Eq. (31) since the change of intervalley FFT-filtering direction only influences $\Delta\mathbf{K}$ as well as the phase $\Delta\mathbf{K} \cdot \mathbf{r}$ but never alters the phase $\theta_{\mathbf{r}}$ in Eq. (31).

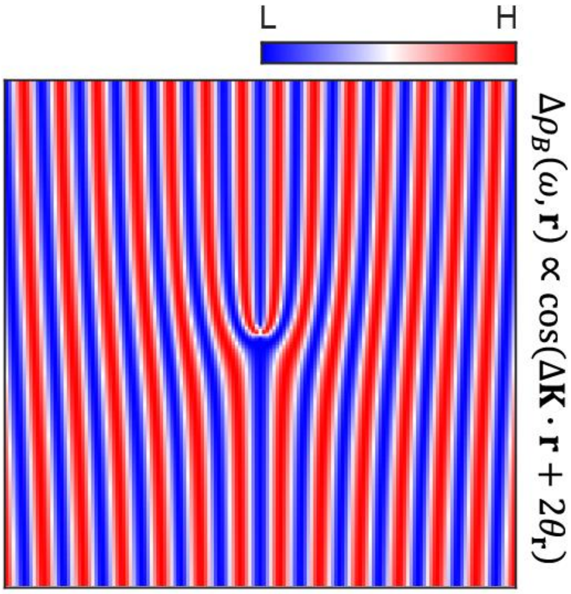

Supplementary Figure 1 | A schematic diagram to show wavefront dislocations in LDOS modulation $\Delta\rho_B(\Delta\mathbf{K}, \mathbf{r})$ on sublattice B with the contribution of pseudospin rotation. The wavelength of cophasal surfaces is 3.7 Å.

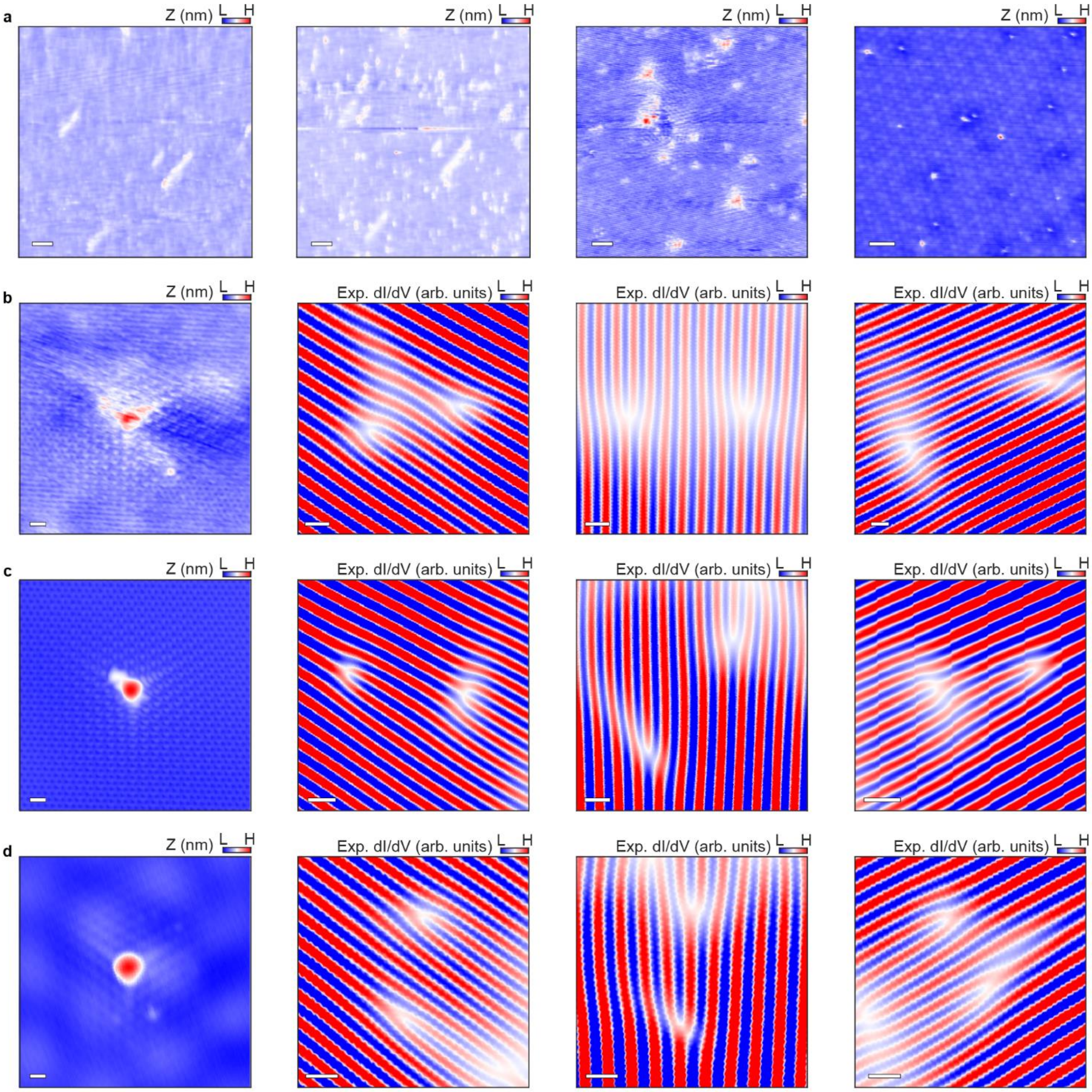

Supplementary Figure 2 | Sample preparation and two wavefront dislocations in three directions. a, Generating Defects. From left to right: typical images of graphene on Ge substrate before (Scale bar: 5 nm) and after STM tip pulses (Scale bar: 5 nm), zoom-in image of defects on Ge substrate (Scale bar: 1 nm), and defect-rich graphene on Cu substrate (Scale bar: 5 nm). **b,** Defects on the semiconducting substrate. The graphene defect and wavefront dislocations on Ge substrate. The defect is generated by the strong STM tip pulse around -4 V to 4 V. The tip pulse cuts off the Ge-H bond and creates hydrogen absorption underneath the monolayer graphene. **c,** Defects on the insulating substrate. Graphene defect and wavefront dislocations on BN substrate. The defect arises intrinsically during the growth process, and defect-rich graphene is subsequently transferred onto the BN substrate for gate-tunable measurements. **d,** Defects on the metallic substrate. One graphene defect on Cu-Ni substrate. The double wavefront dislocations are observed in three directions as well. The panels from **b** to **d** demonstrate the robust and universal nature of the wavefront dislocations across various systems. The scale bars are 0.5 nm for figures **b-d**.

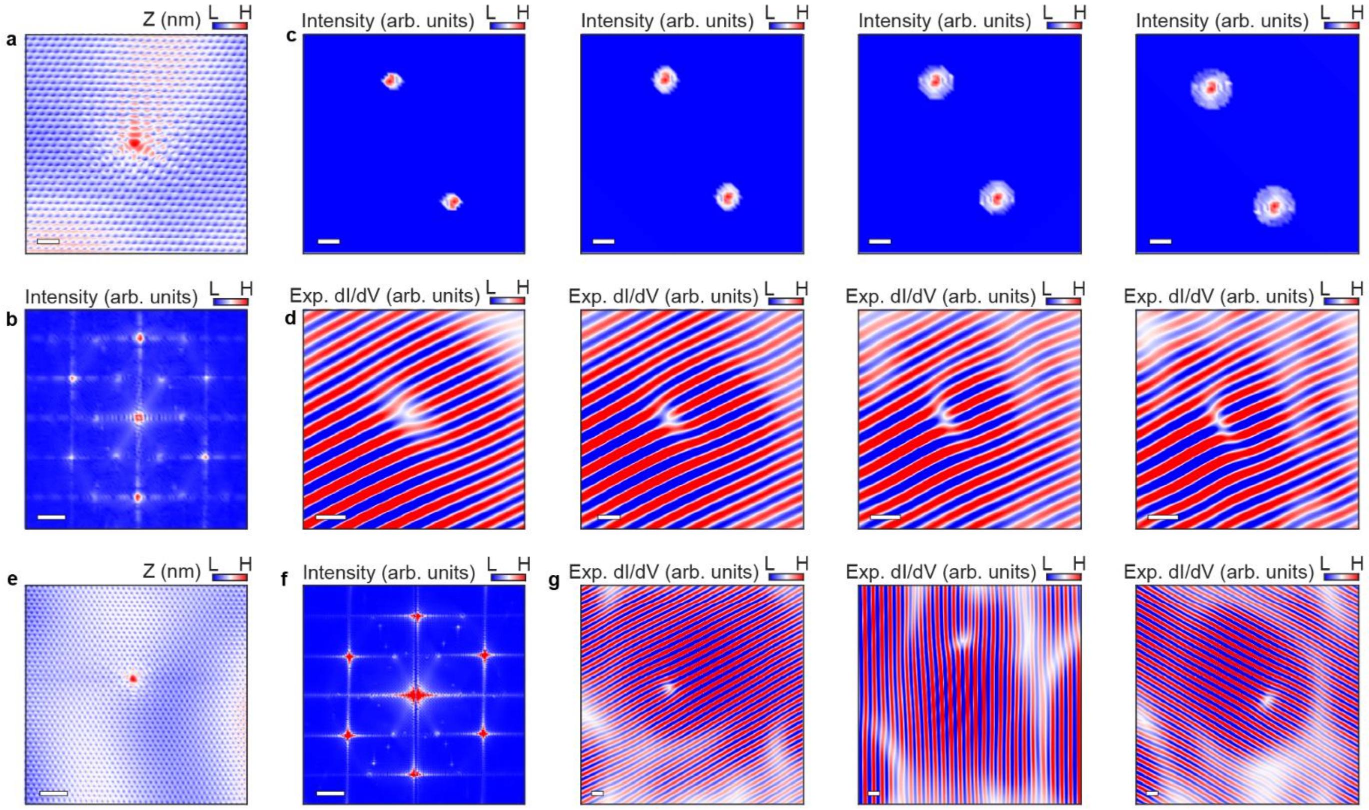

Supplementary Figure 3 | Different filter conditions. a,b, One defect (Scale bar: 0.5 nm, V_b = 0.6 V, setpoint I = 0.3 A) and the corresponding FFT image (Scale bar: 5 nm^{-1}). **c,d,** Upper images display filtered circles of varying sizes, while the lower images represent the corresponding wavefront dislocations, which result from the inverse FFT of the upper images. Scale bar: 10 nm^{-1}. There is always one wavefront dislocation with different filter sizes, which means the single wavefront dislocation is robust and not related to the filter conditions. Scale bar: 0.5 nm. **e-g,** A defect of the same type as above but with a larger image size. The figures display topography window (Scale bar: 1 nm), FFT window (Scale bar: 10 nm^{-1}), and wavefront dislocations window (Scale bar: 0.5 nm) in three directions from left to right, which illustrate that the single wavefront dislocation is still clean and robust in a larger area.

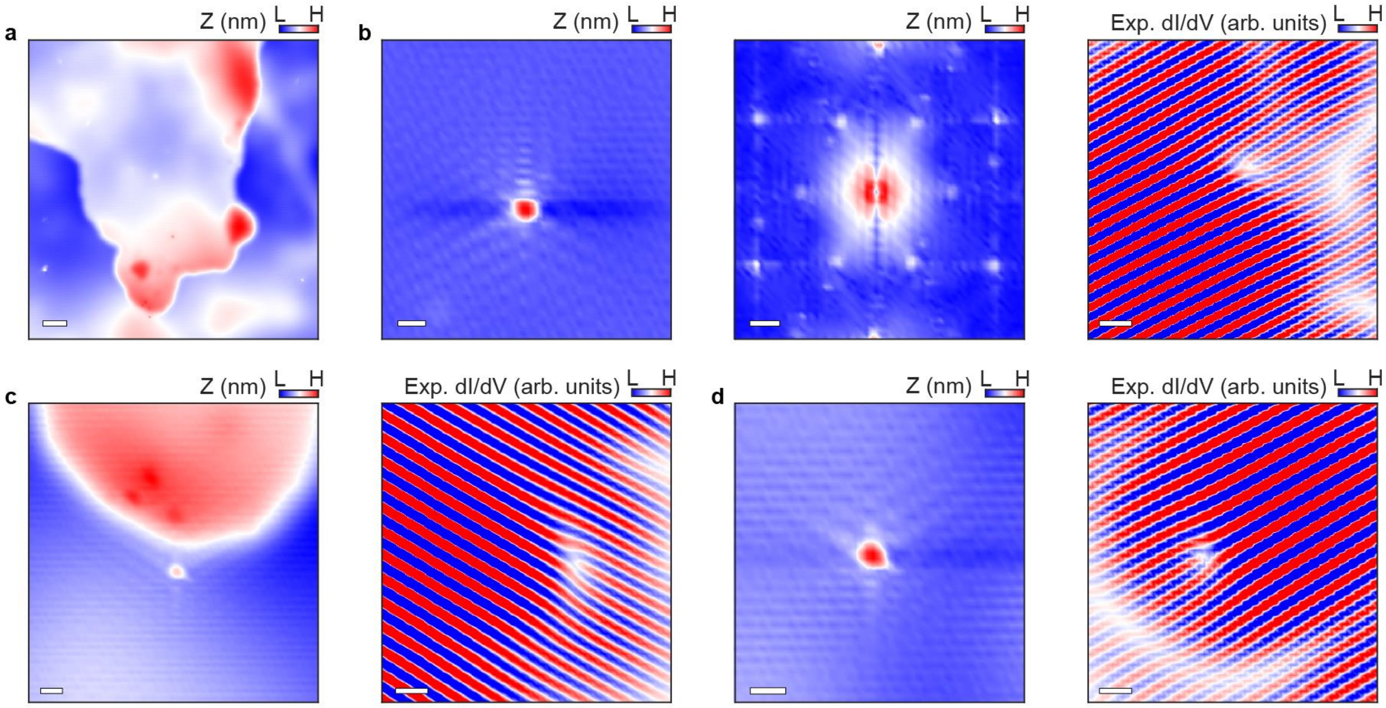

Supplementary Figure 4 | Different defects detected by the same STM tip. a, Large area graphene topography with defects on it. Scale bar: 10 nm. **b-d,** Defects located on and off the step, along with their respective single-wavefront dislocations. The presence of similar single-wavefront dislocations across defects located in different areas indicates that this phenomenon is not caused by local interference from the substrate. The scale bar for defects and wavefronts are 0.5 nm. The scale bar of the FFT image is 5 nm^{-1}.

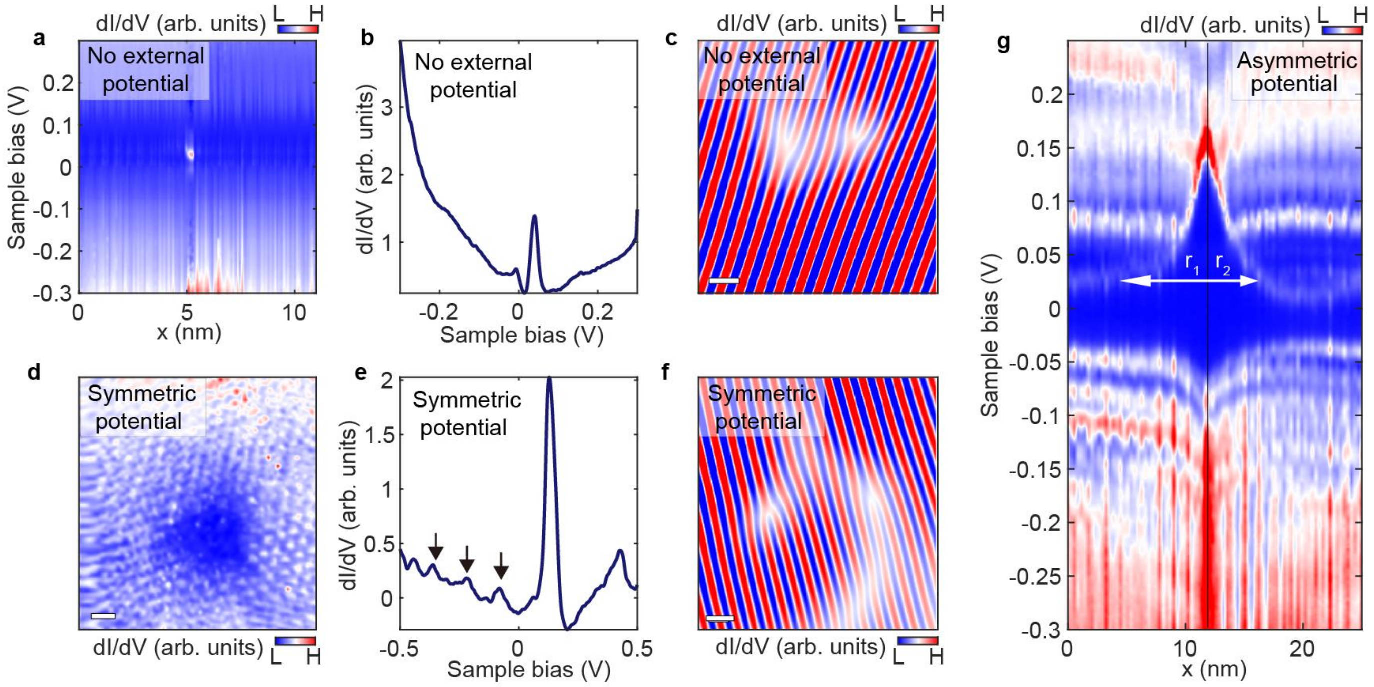

Supplementary Figure 5 | Comparison of different types of potential. a, STS linecut obtained along a hydrogen chemisorption defect without external potential. The defect state, which shows high *dI/dV* intensity, is located at the same energy with the Dirac point of graphene. **b,** STS point spectra extracted from panel **a** around the defect. Only the defect state is observed in the STS. **c,** wavefront dislocations of the same defect as panel **a**, showing two wavefront dislocations in real space**. d-f,** STS map, STS point spectra, and two wavefront dislocations for another hydrogen chemisorption defect. The LDOS in real space exhibits equal intensity surrounding the defect, indicating a relatively symmetric potential in comparison to Fig. 2f. Black arrows in panel **e** with uniform spacing denote the presence of a tip-induced quantum dot effect when detected in the vicinity of the defect region. **g,** STS linecut for the same defect as Fig. 2f under the magnetic field of 8 T. The charging peak shows the asymmetric distance for the same chemical potential, which can reflect the asymmetric tip potential. Scale bar: 0.5 nm.

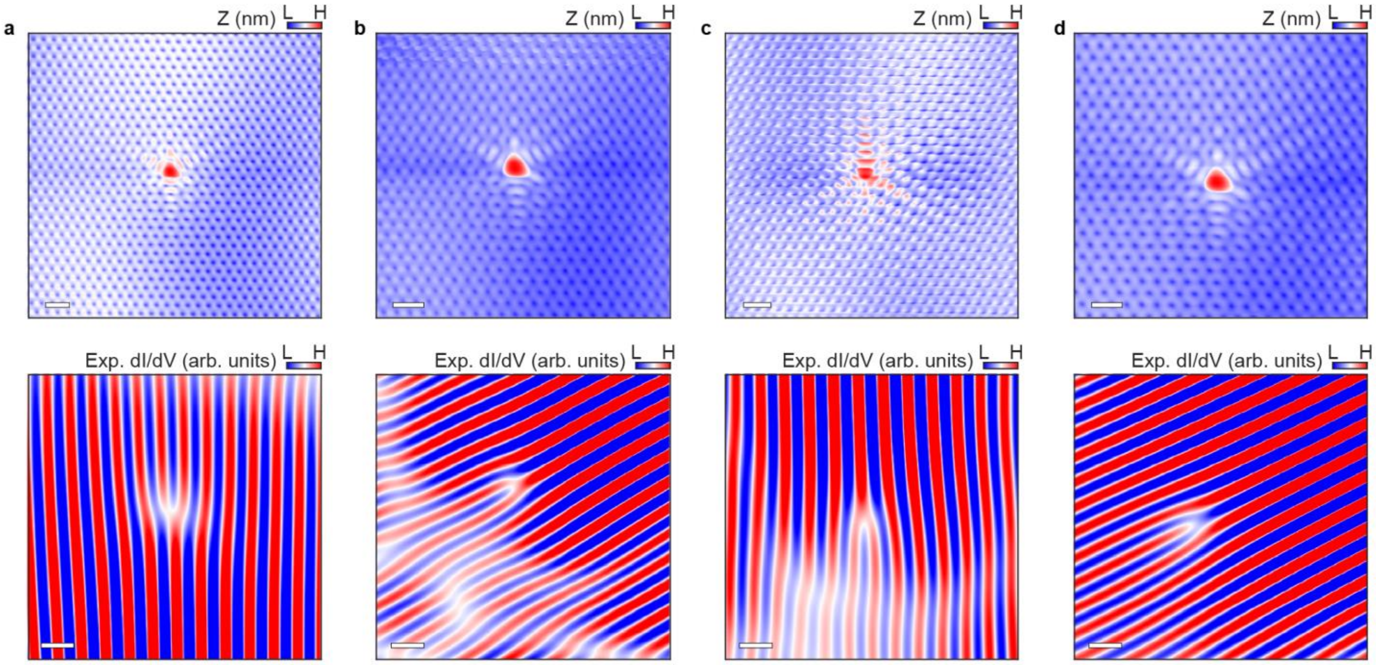

Supplementary Figure 6 | Wavefront dislocations at distinct energies. a-d, Topographic images with different sample bias and setpoint, which are 0.3 V, 0.1 A; 0.5 V, 0.2 A; 0.6 V, 0.3 A; 0.7 V, 0.2 A. The wavefront dislocations at the bottom are derived from the upper defects. Scale bar: 0.5 nm.

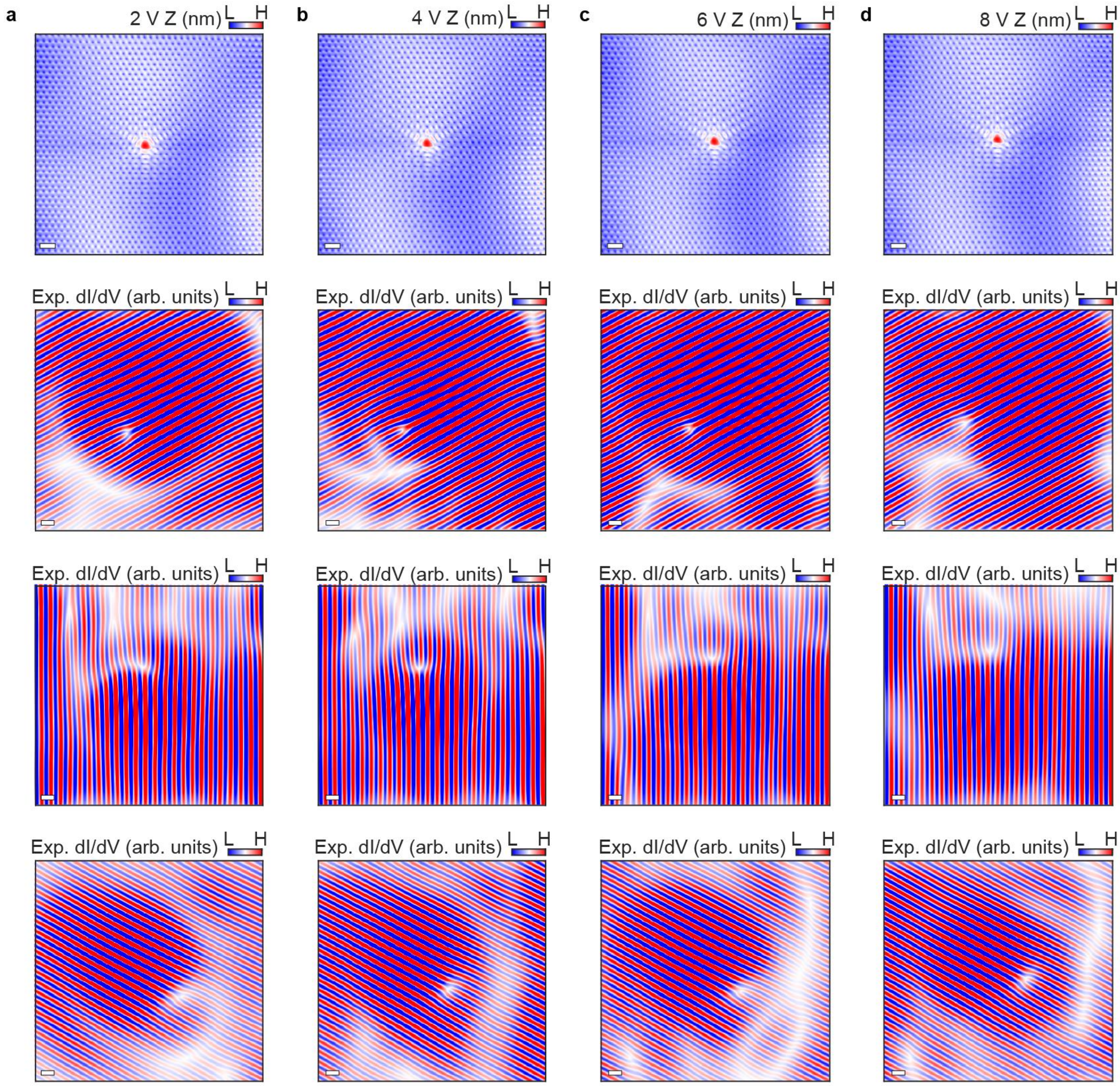

Supplementary Figure 7 | Wavefront dislocations at different back-gate voltages. The sample bias 0.45 V and setpoint 0.2 A are consistent across all images. From the left to right panels, back-gate voltages are 2 V, 4 V, 6 V, and 8 V, respectively. Scale bar: 0.5 nm.

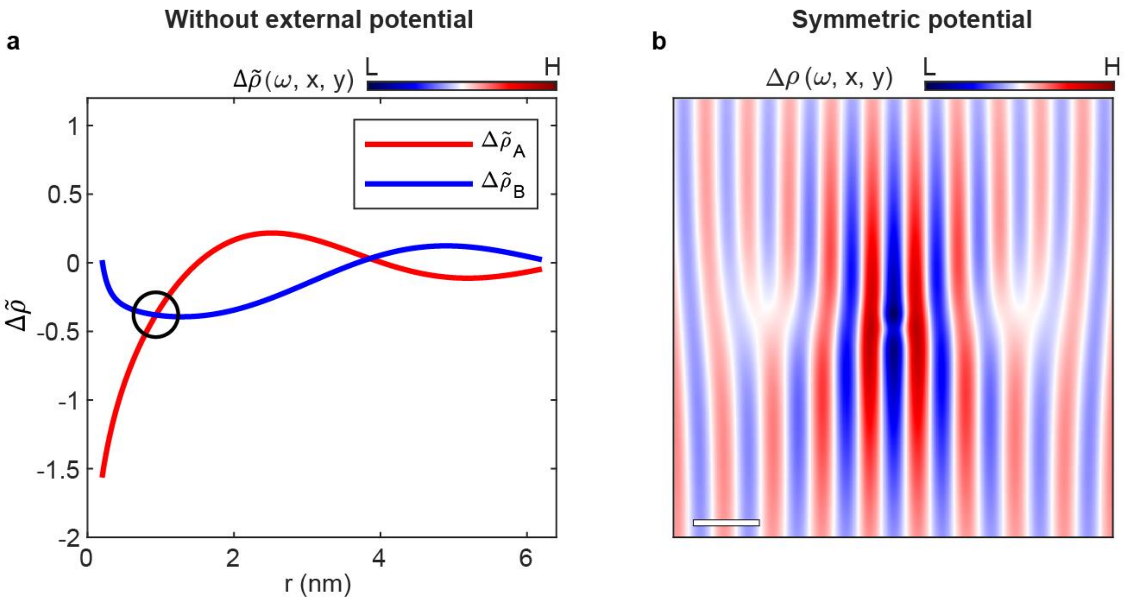

Supplementary Figure 8 | The numerical results of dual-wavefront dislocations. a, The $\Delta\tilde{\rho}_A(\omega,r) \propto Im[\omega^2 H_0^2(\omega r)t(\omega)]$ and $\Delta\tilde{\rho}_B(\omega,r) \propto Im[\omega^2 H_1^2(\omega r)t(\omega)]$ for the energy of $\omega \approx 0.44$ eV. Here r has been converted to nm by multiplying the length unit $r^* =$ 10 nm, and the dark circle denotes the first position for the emergence of additional wavefronts for the calculated result in Fig. 2e, where $\Delta\tilde{\rho}_A(\omega,r) = \Delta\tilde{\rho}_B(\omega,r)$. **b,** The numerically calculated distribution of $\Delta\rho(\omega,x,y)$ with $\omega \approx 0.15$ eV and $\Delta\mathbf{K} = \left(-\frac{4\pi}{3\sqrt{3}a_{cc}}, 0\right)$ under a rotationally symmetric Gaussian potential $V(r,\theta_{\mathbf{r}}) = -V_0 e^{-\frac{r^2}{R^2}}$ with $V_0 \approx 0.25$ eV and R = 18 nm. Scale bar: 0.5 nm.

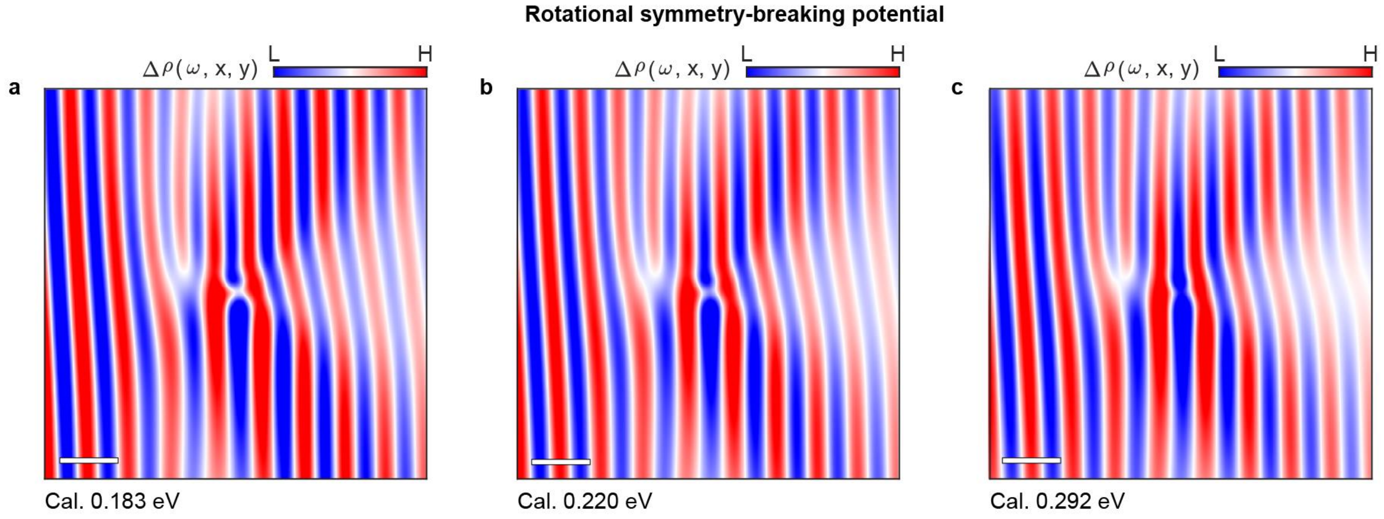

Supplementary Figure 9 | The numerical results of the single wavefront dislocation for different energies. The numerically calculated distribution of $\Delta\rho(\omega, x, y)$ for different energies ω with $\Delta\mathbf{K} = \left(-\frac{4\pi}{3\sqrt{3}a_{cc}}, 0\right)$ under a potential $V(r, \theta_{\mathbf{r}}) = -V_0 e^{-\frac{r^2}{R^2}} + V_1(r, \theta_{\mathbf{r}})$, which breaks the rotation symmetry similar to the calculated results in Fig. 2i. The single wavefront dislocation retains almost unchanged as ω varies. The energies are about 0.183 eV (panel **a**), 0.220 eV (panel **b**), and 0.292 eV (panel **c**). Scale bar: 0.5 nm.

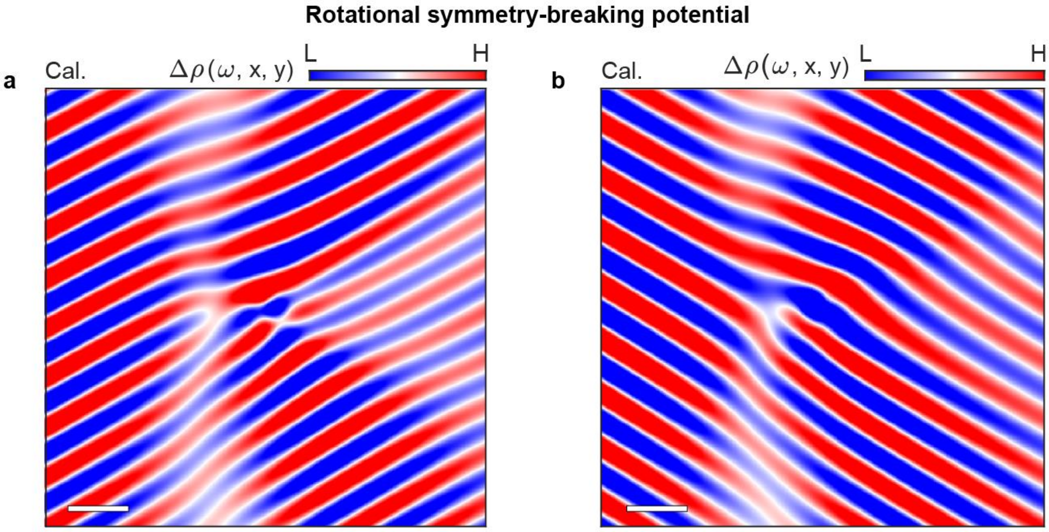

Supplementary Figure 10 | The numerically calculated distribution of $\boldsymbol{\Delta\rho(\omega, x, y)}$ for different filtering directions. The energy is $\omega = 2$ ($\approx$ 0.15 eV) under a potential $V(r, \theta_{\mathbf{r}}) = -V_0 e^{-\frac{r^2}{R^2}} + V_1(r, \theta_{\mathbf{r}})$, which is similar as Supplementary Figure 9a but for two distinct intervalley FFT-filtering directions. **a,** $\Delta\mathbf{K} = \left(\frac{2\pi}{3\sqrt{3}a_{cc}}, -\frac{2\pi}{3a_{cc}}\right)$. **b,** $\Delta\mathbf{K} = \left(\frac{2\pi}{3\sqrt{3}a_{cc}}, \frac{2\pi}{3a_{cc}}\right)$. Scale bar: 0.5 nm.

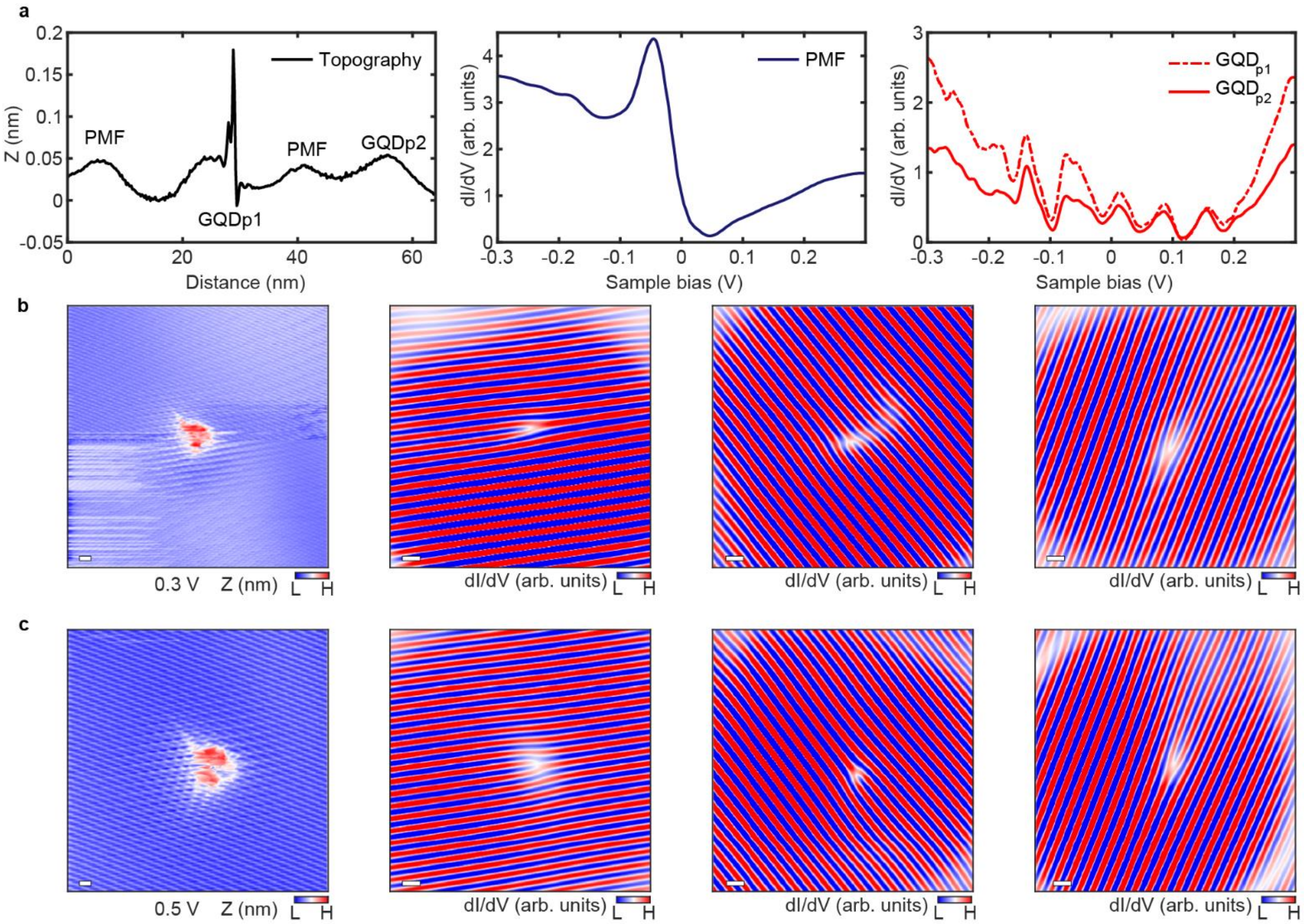

Supplementary Figure 11 | Wavefront dislocations at different energies for the defect on the 1D strained substrate. a, Left panel: the height profile of the periodic strain structure. Middle panel: the strain-induced zeroth pseudo-Landau level spectrum. Right panel: pseudomagnetic confined states with an energy spacing of about 0.7 V. PMF represents the region where the spectrum shows pseudomagnetic field features. GQD represents the region where the spectrum shows graphene quantum dot features. **b,c,** Single-wavefront dislocations are observed at different energies, i.e., 0.3 V and 0.5 V, and different filtering directions. Scale bar: 0.5 nm.

Supplementary References

1. A. H. Castro Neto, F. Guinea, N. M. R. Peres, K. S. Novoselov, and A. K. Geim, The electronic properties of graphene, *Rev. Mod. Phys.* **81**, 109-162 (2009).
2. C.-H. Park and N. Marzari, Berry phase and pseudospin winding number in bilayer graphene, *Phys. Rev. B* **84**, 205440 (2011).
3. Z. Hou, Y.-F. Zhou, X. C. Xie, and Q.-F. Sun, Berry phase induced valley level crossing in bilayer graphene quantum dots, *Phys. Rev. B* **99**, 125422 (2019).
4. C. Dutreix, H. González-Herrero, I. Brihuega, M. I. Katsnelson, C. Chapelier, and V. T. Renard, Measuring the Berry phase of graphene from wavefront dislocations in Friedel oscillations, *Nature* **574**, 219-222 (2019).
5. C. Dutreix and M. I. Katsnelson, Friedel oscillations at the surfaces of rhombohedral N-layer graphene, *Phys. Rev. B* **93**, 035413 (2016).
6. Y. Zhang, Y. Su, and L. He, Local Berry Phase Signatures of Bilayer Graphene in Intervalley Quantum Interference, *Phys. Rev. Lett.* **125**, 116804 (2020).
7. J. Friedel, XIV. The distribution of electrons round impurities in monovalent metals, *Philos. Mag.* **43**, 153 (1952).
8. Nye, J. F. & Berry, M. V. Dislocations in wave trains. *Proc. R. Soc. Lond. A* **336**, 165–190 (1974).
9. Y.-C. Zhuang and Q.-F. Sun, wavefront dislocations in graphene systems revealed by transport measurement, *Phys. Rev. B* **107**, 235423 (2023).
10. Y.-N. Ren, Q. Cheng, S.-Y. Li, C. Yan, Y.-W. Liu, K. Lv, M.-H. Zhang, Q.-F. Sun, and L. He, Spatial and magnetic confinement of massless Dirac fermions, *Phys. Rev. B* **104**, L161408 (2021).
